# Effects of Digital Health Interventions on Breastfeeding Rates, Self-Efficacy, and Knowledge: Systematic Review and Meta-Analysis

**DOI:** 10.2196/92184

**Published:** 2026-08-12

**Authors:** Jiahe Sun, Yu Wang, Shuang Hu, Yajie Ding, Congshan Pu, Danni Song, Jiaai Xia, Chunjian Shan

**Affiliations:** 1 School of Nursing Nanjing Medical University Nanjing, Jiangsu China; 2 Department of Obstetrics Women’s Hospital of Nanjing Medical University (Nanjing Women and Children’s Healthcare Hospital) Nanjing, Jiangsu China

**Keywords:** breastfeeding, DHIs, digital health interventions, digital interventions, eHealth, maternal health, meta-analysis, mHealth, systematic review

## Abstract

**Background:**

The current global status of breastfeeding is marked by both progress and challenges. Digital health interventions (DHIs) have emerged as a promising strategy for improving breastfeeding practices, yet evidence regarding their impact on breastfeeding outcomes remains limited.

**Objective:**

This study aimed to evaluate the impact of DHIs on breastfeeding practices and outcomes.

**Methods:**

Following PRISMA (Preferred Reporting Items for Systematic Reviews and Meta-Analyses) guidelines, we searched PubMed, Web of Science, the Cochrane Library, Embase, CINAHL, Scopus, IEEE Xplore, and gray literature databases, from inception to April 2026. We included randomized controlled trials (RCTs) and quasi‑experimental studies that enrolled pregnant women or lactating mothers using DHIs vs standard care, waiting lists, or placebo. Outcomes were exclusive breastfeeding (EBF) rates, self‑efficacy, and knowledge. Studies recruiting mothers with infectious diseases or severe substance use disorders, non‑English publications, unavailable full texts or data, animal studies, letters, conference proceedings, and studies rated as “high risk” were excluded. Study quality was evaluated using the Cochrane Risk of Bias Tool version 2 and the Risk of Bias in Nonrandomized Studies of Interventions tool. Evidence certainty was assessed using GRADE (Grading of Recommendations, Assessment, Development, and Evaluation). We performed the analysis using Review Manager (version 5.4) and R software (version 4.6.0), estimated using the Hartung-Knapp-Sidik-Jonkman random-effects model.

**Results:**

A total of 52 studies involving 11,704 participants were included. DHIs improved EBF rates (at <3 months: relative risk [RR] 1.33, 95% CI 1.18-1.50, 95% prediction interval [PI] 0.85-2.08; *P*<.001; *I*^2^=84%; at 3-6 months: RR 1.46, 95% CI 1.22-1.75, 95% PI 0.83-2.55; *P*<.001; *I*^2^=75%; at ≥6 months: RR 1.64, 95% CI 1.27-2.11, 95% PI 0.74-3.62; *P*<.001; *I*^2^=86%) and breastfeeding self-efficacy (standardized mean difference 0.67, 95% CI 0.31-1.03, 95% PI –1.17 to 2.51; *P*<.001; *I*^2^=95%). No statistically significant effects were observed for breastfeeding knowledge. The subgroup analysis suggested that antenatal intervention delivery; intervention duration ≥3 months, developing-country setting; and participants who were preterm parturients, adult women, or mothers undergoing cesarean delivery could improve the EBF rate. In contrast, larger effect sizes for breastfeeding self-efficacy were observed among women with intervention duration <3 months, full-term delivery, and singleton pregnancy. Among RCTs, 12 were low risk, and 30 had some issues; among the quasi-experimental studies, 3 were low risk, and 7 were moderate risk. Evidence certainty ranged from low to moderate.

**Conclusions:**

DHIs show promise for improving EBF rates and breastfeeding self-efficacy. Compared with previous reviews, this review provides a more comprehensive evaluation of DHIs and identifies potential effect modifiers through subgroup analyses, offering updated evidence for digital breastfeeding support. However, moderate risk of bias, substantial heterogeneity, wide 95% PI, and low to moderate GRADE certainty warrant cautious interpretation. Further high-quality studies are needed to confirm effectiveness and optimize implementation of DHIs in clinical practice.

**Trial Registration:**

PROSPERO CRD420251233352; https://www.crd.york.ac.uk/PROSPERO/view/CRD420251233352

## Introduction

Breastfeeding is recognized by the World Health Organization (WHO) as the best source of nutrition for infants and young children [1,2]. Epidemiological studies of mothers indicate that breastfeeding promotes postpartum recovery and reduces multiple disease risks for mothers and infants [3-5]. The WHO recommends EBF from birth to 6 months of age, with continued breastfeeding until 2 years of age or beyond [6,7]. By 2024, nearly half (48%) of infants under 6 months of age were exclusively breastfed, bringing the global overall rate close to the 50% target set for 2025 [8]. Building on this progress, the WHO has raised the target to 60% and extended the deadline to 2030 [9]. However, in low- and middle-income countries, the rate of EBF for infants under 6 months is only 37% [10,11]. Conversely, high-income countries exhibit shorter breastfeeding durations [10,11]. Regardless of development level, rates of early initiation of breastfeeding and EBF within the first 6 months of life remain below 60% in all countries, with significant variations between nations [10,11]. Therefore, interventions are needed to improve this outcome.

Conventional in-person breastfeeding interventions are subject to multiple implementation limitations, including limited access to health care services, poor adherence to standardized intervention protocols, and insufficient individualized support [12]. Pregnant and postpartum women often encounter unique barriers such as mobility restrictions, geographical constraints, and inadequate resources [13,14]. Offline health education experiences limited timeliness and fails to deliver long-term continuous feeding monitoring and tailored guidance, which generally reduces maternal intervention adherence and hinders sustained EBF [15,16]. Furthermore, standardized group-based health education cannot accommodate the distinct individual needs of diverse women regarding breastfeeding knowledge, psychological well-being, and feeding practices [17]. Consequently, there is a growing interest in exploring novel intervention approaches and alternative strategies to address these persistent challenges.

In recent years, digital health interventions (DHIs) have rapidly integrated into the health care sector as a transformative technology, offering highly promising new avenues for improving breastfeeding outcomes [18,19]. DHIs refer to personalized medical and health management services that combine health care with information and communication technologies. Furthermore, digital health integrates various digital technologies in the medical field, such as the Internet of Things, AI, extensive data analysis, and robotics [20]. The WHO defines DHIs as “an independent technological function or capability designed to achieve specific objectives and address challenges in health systems” [21]. At present, DHIs are delivered via a wide range of digital technological carriers, including mobile apps, telemedicine, video, virtual reality (VR), wearable devices, and AI. Their target populations have expanded from healthy women with term delivery to mother-infant dyads with special clinical needs, such as women experiencing preterm birth and those receiving cesarean delivery. DHIs have been widely used in breastfeeding, and their potential effectiveness has been preliminarily validated. For instance, mobile apps providing structured educational content and AI-driven SMS text message counseling have been shown to promote EBF and enhance maternal self-efficacy [22,23], while VR-based programs incorporating relaxation techniques have demonstrated benefits in reducing maternal anxiety and improving lactation outcomes [24,25].

To systematically evaluate the efficacy of different types of DHIs for breastfeeding support, this review classifies DHIs into 3 major categories according to the general technical framework in the digital health field [26], based on their technical carrier and core functional characteristics. The 3 categories are eHealth, mobile health (mHealth), and digital devices. eHealth refers to the use of emerging information and communication technologies, such as the internet, to improve or facilitate health and health care services [27], including video [28], websites [29], telemedicine [30,31], and even AI [32,33]. By leveraging these platforms, eHealth enables professional breastfeeding guidance to transcend geographical and temporal barriers, offering pregnant and postpartum women holistic, personalized, and sustained support. In breastfeeding practice, eHealth modalities encompass educational video courses, dedicated web platforms [34], remote online lactation consultations [35], chatbot-based counseling [36], and machine learning-driven prediction of EBF outcomes [37]; mHealth refers to health care and public health practices supported by mobile devices or mobile communications [38,39], with technical carriers including SMS text messaging [40] and mobile apps [41]. mHealth technologies feature low technical barriers and real-time reminder delivery, making them widely adopted interventions for breastfeeding support. Common applications include SMS-based breastfeeding reminders and mobile apps offering breastfeeding guidance [42,43]; digital devices refer to physical hardware components used to process, store, or transmit digital data for health purposes [44], including smartphones [45], tablets [46], wearable devices [47], and sensors. Their core function lies in enabling objective monitoring and real-time feedback on breastfeeding behaviors. In breastfeeding contexts, these devices are used for continuous tracking of feeding frequency, milk output, and infant latching status via lactation wearables [48], as well as for delivering VR-based simulations to enhance maternal breastfeeding knowledge and self-efficacy [49]. This 3-category framework provides a structured basis for comparing how different digital intervention modalities may affect breastfeeding outcomes.

Previous systematic reviews have evaluated a range of DHIs for breastfeeding support, yet considerable uncertainty remains regarding their overall effectiveness across different breastfeeding outcomes. Existing reviews have typically focused on a single type of DHI or a single breastfeeding outcome [50-52], and evidence comparing intervention effects across different DHI categories or implementation timing remains limited [53,54]. Furthermore, findings from randomized controlled trials (RCTs) on the effects of DHIs on breastfeeding self-efficacy have been inconsistent [55-57]. This conflicting evidence underscores the need for an updated comprehensive meta-analysis to evaluate the effectiveness of various DHIs across multiple breastfeeding outcomes and to explore potential sources of heterogeneity.

Therefore, this systematic review and meta-analysis aims to comprehensively evaluate the effects of DHIs on EBF rates, breastfeeding self-efficacy, and breastfeeding knowledge. Compared with previous systematic reviews, this study compares different DHI modalities and examines how intervention characteristics, national income levels, and population characteristics may moderate intervention effects, providing evidence-based support for developing targeted digital health strategies for breastfeeding.

## Methods

### Overview

This systematic review and meta-analysis report adheres to the 2020 PRISMA (Preferred Reporting Items for Systematic Reviews and Meta-Analyses) statement (Multimedia Appendix 1) and PRISMA-S (Preferred Reporting Items for Systematic Reviews and Meta-Analyses literature search extension) to enhance transparency (Multimedia Appendix 2) [58,59]. The review protocol has been registered with PROSPERO (International Prospective Register of Systematic Reviews; CRD420251233352).

### Search Strategy

We conducted a search of PubMed, Web of Science, the Cochrane Library, Embase, CINAHL, Scopus, IEEE Xplore, and gray literature by October 2025. The search was updated in April 2026 to identify any newly published relevant studies. First, the search strategy and search terms were finalized through group discussion. Subsequently, preliminary database searches were performed, and the strategy and terms were further revised based on the retrieved results. Formal searches were then conducted in each database using the finalized search strategy and keywords. In addition, we manually reviewed the reference lists of included studies to identify any potentially omitted studies. The search strategy combined subject headings and free-text terms. The main subject terms included breastfeeding, AI, SMS text messaging, wearable sensors, telemedicine, mobile apps, internet-based interventions, RCTs, and non-RCTs. This review did not use published search filters. We did not adopt or reuse search strategies from previously published systematic reviews during our literature search. We manually screened lists of eligible studies and cited references via Google Scholar to identify additional relevant literature. We searched the US clinical trials registry (ClinicalTrials.gov) and the Chinese clinical trials registry to identify eligible studies. For studies where full-text articles and data were unavailable, we contacted the corresponding authors to attempt to obtain the full text. Given the availability of academic resources and the language proficiency of the research team, only studies published in English were included, with no publication date restrictions. The complete search strategy is available in Multimedia Appendix 3.

### Eligibility Criteria

We included the following studies:

- Population: pregnant women or breastfeeding mothers
- Interventions: DHIs as classified by the WHO, including eHealth (internet-based interventions such as videos, websites, telemedicine, and AI), mHealth (text messages and apps), and digital devices (VR, wearable devices, etc)
- Control groups: standard care, waiting list, or placebo control groups
- Outcomes: EBF rates, breastfeeding self-efficacy, and breastfeeding knowledge levels
- Study designs: RCTs and quasi-experimental designs. Study designs were chosen to balance bias control with evidence volume. Recognizing RCTs as the gold standard [60], we also included quasi-experimental studies to address the complexity of DHIs and the current scarcity of AI-focused breastfeeding research. Defined by nonrandomized allocation while still including a comparison group (eg, matched or temporal grouping), these studies serve as robust evidence substitutes when RCTs are limited [61-63]. In contrast, observational and qualitative designs were excluded to minimize confounding risk and ensure quantifiable results [64].

The following studies were excluded: studies involving mothers with infectious diseases (eg, HIV) or severe substance use disorders, studies published in languages other than English, studies for which full-text articles were unavailable, studies with missing or unavailable data, animal studies, letters or conference proceedings, and studies rated as “high risk” [65].

### Study Selection and Data Extraction

All retrieved articles were imported into EndNote software (Clarivate Analytics). After removing duplicates, two researchers (JS and YW) independently reviewed the article titles and abstracts and subsequently read the full texts to determine eligibility based on the inclusion criteria. Disagreements were resolved through discussion with a third researcher (SH), who made the final decision.

Two researchers (JS and YW) independently extracted data using a customized data extraction form. The details extracted included but were not limited to study details (eg, title, authors, year, and country), study design (study type, objective, measurement tools, and inclusion/exclusion criteria), participant characteristics (sample size, age, gestational age, number of fetuses, mode of delivery, etc), breastfeeding outcomes (EBF rates, breastfeeding self-efficacy, and breastfeeding knowledge), and intervention characteristics and dose (intervention type, period, frequency, and duration).

There were no issues of double counting in this meta-analysis, as the participants in each included study constituted independent samples [66]. Therefore, for studies with multiple intervention groups, we did not combine the interventions; instead, we extracted data only from the groups directly relevant to the study objectives for analysis. For studies reporting the same outcome at multiple time points, to ensure consistency of results and minimize the potential influence of time on the findings, we included only data from the first time point following intervention in the meta-analysis [66].

### Risk of Bias Assessment and Certainty of Evidence

Quality assessment was conducted during the full-text screening phase to inform study eligibility. Two researchers (JS and YW) independently assessed the quality of RCTs using the Cochrane Risk-of-Bias Tool version 2 (RoB2) [63,67], which evaluates random sequence generation, allocation concealment, participant and staff blinding, outcome assessor blinding, missing outcome data, selective reporting, and other sources of bias. We used the Risk of Bias in Nonrandomized Studies of Interventions (ROBINS-I) tool to assess the quality of quasi-experimental studies [68]. Based on the overall assessment results of ROBINS-I, the risk level for each study was categorized as “critical risk, serious risk, moderate risk, low risk, or insufficient information to assess risk of bias.” During the screening phase, we first assessed the methodological quality of included studies using the RoB2 [67,69] and ROBINS-I [68]. Studies with at least one item or an overall bias risk rated as “high” were excluded. To minimize the systematic impact of biases such as selective reporting, inadequate blinding, and incomplete data on effect estimates, which could compromise the robustness of the pooled results, these studies were excluded from the primary analysis. Risk of bias results for the included studies were visualized using Robvis [70].

Two authors (JS and YW) independently scored the certainty of evidence using the GRADE (Grading of Recommendations, Assessment, Development, and Evaluation) criteria [71,72]. Disagreements were resolved by consulting a third researcher (SH).

### Statistical Analysis

We conducted the meta-analysis using Review Manager (version 5.4; Cochrane Collaboration) and R software (version 4.6.0; R Foundation for Statistical Computing). The restricted maximum likelihood estimator was used to estimate the between‑study variance (τ^2^) [73]. The Hartung-Knapp-Sidik-Jonkman random-effects model was used to reanalyze the data and estimate CIs [74]. EBF rates were estimated using relative risk (RR) and 95% CI, with data pooled across three time periods: <3 months, 3-6 months, and ≥6 months. Breastfeeding self-efficacy and breastfeeding knowledge levels were estimated using the standardized mean difference (SMD) and 95% CI. Due to significant differences in participant characteristics, intervention methods, and outcome measurement methods, the data were analyzed using a random-effects model. To assess statistical heterogeneity, τ^2^ was calculated. We also derived Cochran’s Q test and the *I*^2^ statistic to jointly quantify the extent and significance of observed heterogeneity. For results with a sufficient number of studies (n≥3), we calculated a 95% prediction interval (PI) to quantify the practical clinical significance of heterogeneity [75]. Sensitivity analyses were conducted by sequentially excluding all included studies. Potential small-sample effects were assessed using funnel plots combined with the Egger regression test [76].

Subgroup analyses were conducted to explore potential sources of heterogeneity. Prespecified subgroup analyses were conducted to identify potential sources of between-study heterogeneity and examine whether intervention characteristics moderate the effects of DHIs on breastfeeding-related outcomes. Prespecified subgroup analyses were performed stratified by (1) DHIs type (mHealth: SMS text messaging and apps; eHealth: internet-based, websites, and videos; or digital devices: wearables and VR) [77]; (2) intervention stage (prenatal vs postnatal); (3) intervention duration (<3 months vs ≥3 months); (4) intervention frequency (<3 times/week vs ≥3 times/week); in addition, to further investigate the substantial heterogeneity observed in the pooled analyses, we conducted supplementary exploratory subgroup analyses stratified by (5) national income level (developed countries vs developing countries, with grouping standards following the official country income classification criteria released by the World Bank); and (6) population characteristics: gestational age (term vs preterm), age (adults vs adolescents), number of fetuses (singleton vs multiple), mode of delivery (vaginal vs cesarean). All findings from exploratory subgroup analyses should be interpreted with caution and regarded only as exploratory evidence to generate new research hypotheses.

### Ethical Considerations

This meta-analysis was a secondary analysis of published literature and did not require ethics approval. As there is no direct contact with subjects and no individual-level data are collected, informed consent is waived. This analysis strictly adheres to the principles of patient privacy protection: all data used are aggregate statistics or fully anonymized data that contain no personally identifiable information (eg, names, initials, and hospital numbers), such information having been omitted as it is not essential for scientific purposes. All original studies included in this meta-analysis obtained their own ethical approval and informed consent from subjects within their respective research contexts.

## Results

### Search Selection

**Figure 1:**
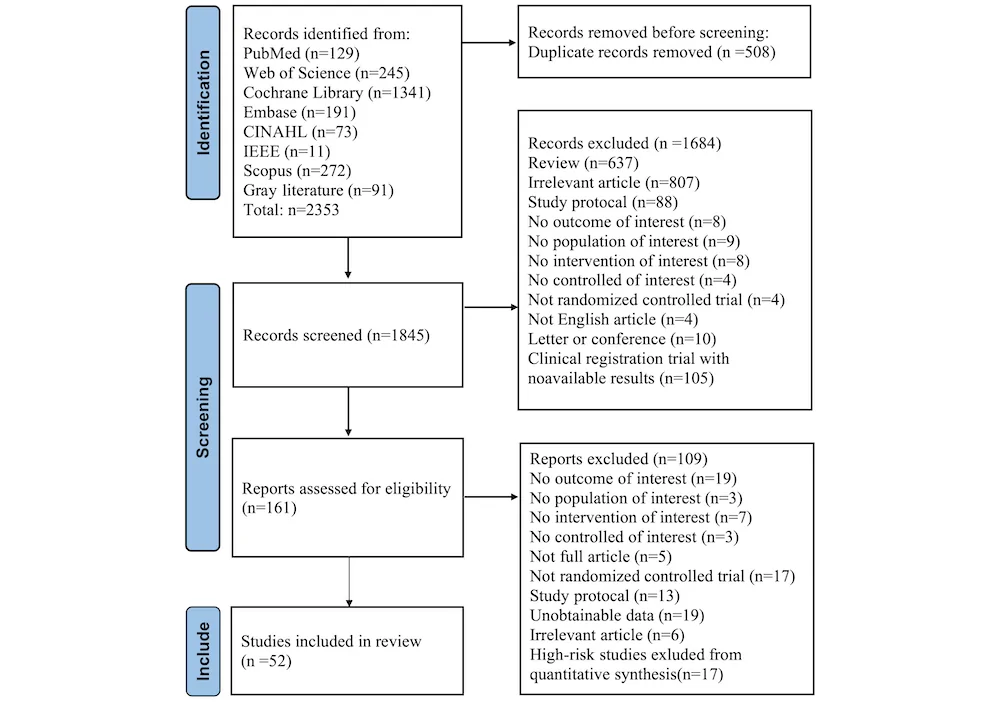
Flowchart of the study selection process, following the 2020 PRISMA (Preferred Reporting Items for Systematic Reviews and Meta-Analyses). Screening and inclusion for a systematic review and meta-analysis of randomized controlled trials and nonrandomized controlled trials.

Initially, 2353 articles were identified from the databases. After excluding 508 duplicate articles, 2 researchers independently screened the titles and abstracts of the remaining 1845 articles. Of these, 1684 articles were excluded for irrelevance. Full texts of the remaining 161 articles were retrieved and assessed for eligibility. Studies rated as high risk of bias were excluded during this stage. Ultimately, 52 articles were included in the meta-analysis [34,35,42,43,49,56,57,78-122]. The literature screening process is shown in Figure 1.

### Study Characteristics

The 52 included studies were published between 2015 and 2026 [34,35,42,43,49,56,57,78-122], with 15 conducted in Turkey [34,35,49,78,81,83,84,86,87,97-99,101,108,121], 8 in China [43,56,90,94-96,119,120], 6 each in the United States [79,85,92,93,100,114] and Iran [82,88,111,113,115,122], 2 each in India [91,109] and Saudi Arabia [80,110], and 1 each in Nigeria [116], Japan [106], Malaysia [103], Colombia [104], Spain [104], Kenya [107], Uganda [105], Vietnam [89], Myanmar [57], Australia [118], Israel [102], Ethiopia [42], and Indonesia [112]. This study included 42 RCTs [34,35,42,43,49,56,57,78,79,81,83-85,87, 89-95,97,99-105,107,109,110,113-122] and 10 quasi-experimental studies [80,82,86,88,96,98,106,108,111,112]. Among these, 3 studies recruited adolescent mothers [34,87,116]; 3 studies included mothers of critically preterm infants [100,110,111]; 3 studies included women who underwent cesarean section [89,97,121]; and 2 studies included women with multiple pregnancies [103,109]. The total sample size for this study was 11,704, with individual trial sample sizes ranging from 23 to 1717 participants. Interventions lasted from 1 day to 9 months, with a frequency ranging from once a week to 10 times a week.

Among the 52 included studies, DHIs types could be categorized into 3 groups: mHealth (n=30) [42,43,56,57,78,81-83,85,89,90,92-96, 100,102,103,105,107,109,112-118,121], eHealth (n=17) [34,35,79,80,84,86-88,91,97-99,101,106,108,110,119], and digital devices (n=5) [49,104,111,120,122]. A total of 30 studies primarily implemented interventions during the prenatal period [34,35,42,43,49,56,57,80,81,84,85,89,91,92,94-96,98,101,103-106, 108,112,114,117-120], while the remaining 22 initiated interventions postpartum [78,79,82,83,86-88,90,93,97, 99,100,102,107,109-111,113,115,116,121,122]. Two studies were 3-arm RCTs [85,118]. For studies with multiple intervention groups, we extracted data only from the groups directly relevant to the study objectives for analysis. To ensure consistency of results and minimize the potential impact of time on the findings, we adopted the methodological protocol used in a previous meta-analysis [123]. The outcomes we examined included EBF rates (n=37) [34,42,43,56,57,78-85,87,89-97, 99,100,102,104-106,109,110,112,114,117-120], breastfeeding self-efficacy (n=28) [34,35,49,81,83,85-88,90,91, 93,97,98,101,103,104,106,108-111,113-115,119,121,122], and breastfeeding knowledge (n=30) [86,94,103,107,115,116]. Although the outcome assessment tools varied across studies, all were valid scales, and data collection was successfully conducted. A total of 24 studies used the Brief Breastfeeding Self-Efficacy Scale-Short Form [34,35,81,83,86-88,90,91,93, 97,98,103,104,106,108-111,113,114,119,121,122]. Two studies used the full Breastfeeding Self-Efficacy Scale [85,115], 2 used the Prenatal Breastfeeding Self-Efficacy Scale [49,101], 4 used a self-designed Breastfeeding Knowledge Questionnaire [94,103,107,116], 1 used a Knowledge, Attitudes, and Practices questionnaire to assess this outcome [115], and 1 used the Breastfeeding and Breastfeeding Knowledge Test [86]. Detailed information is presented in (Multimedia Appendix 4) [34,35,42,43,49,56,57,78-121,124].

### Risk of Bias Assessment

**Figure 2:**
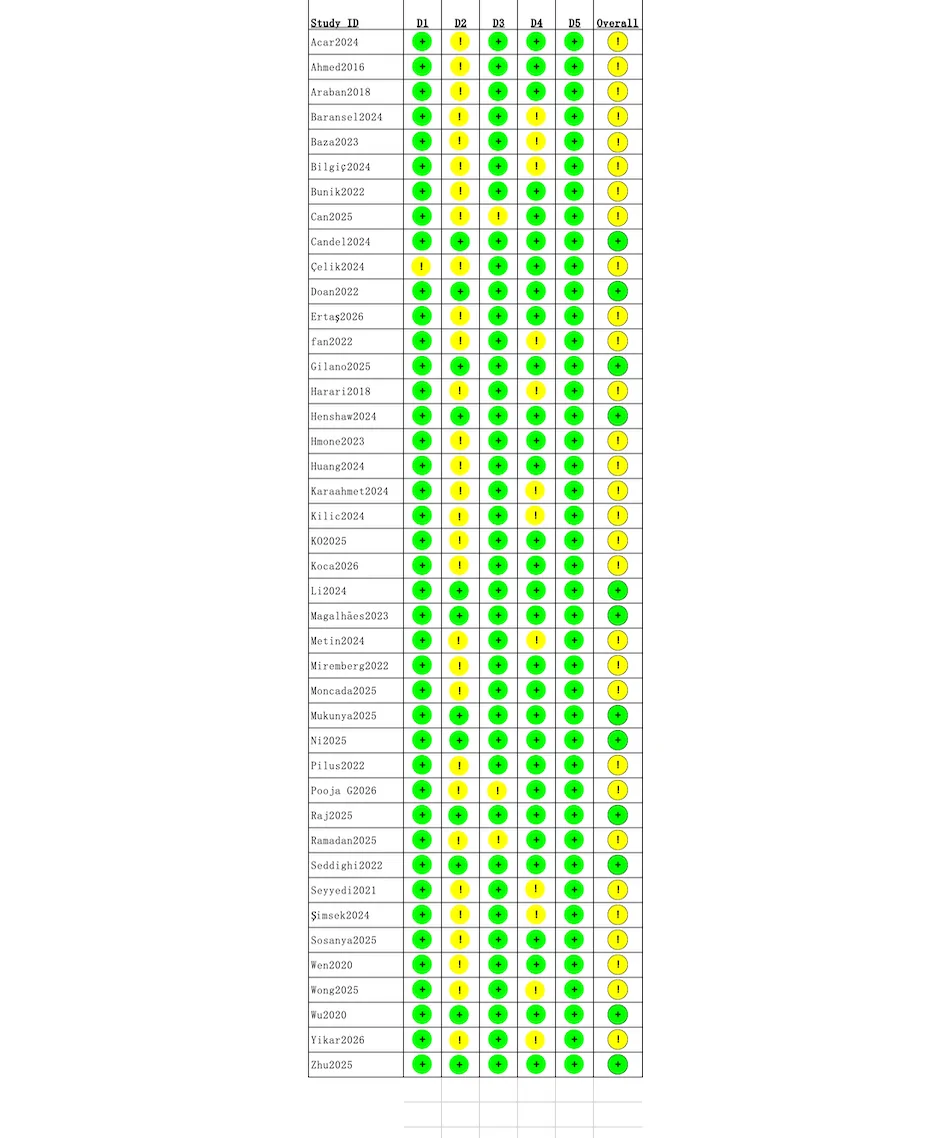
Visualization of the risk of bias of randomized controlled trials (n=42).

Among the included RCTs, 12 studies were classified as low risk [42,43,56,89,93,95,100,105,109,113,117,120] and 30 had some concerns [34,35,49,57,78,79,81,83-85,87,90-92,94,97,99, 101-104,107,110,114-116,118,119,121,122]. Most studies showed some concerns for deviations from intended interventions, primarily because blinding of participants and personnel was impractical in DHIs, and adherence to the intervention could not always be fully ensured. Three of these studies had dropout rates exceeding 5%, raising concerns about attrition bias [34,91,110]. Overall, 12 of the studies were rated as having some concerns because the outcomes were self-reported and outcome assessors were not blinded [35,83,84,90,92,97,101,114,115,119,121,122] (Figure 2 [34,35,42,43,49,56,57,78,79,81,83-85,87,89-95,97,99-105,107,109,110,113-122]).

**Figure 3:**
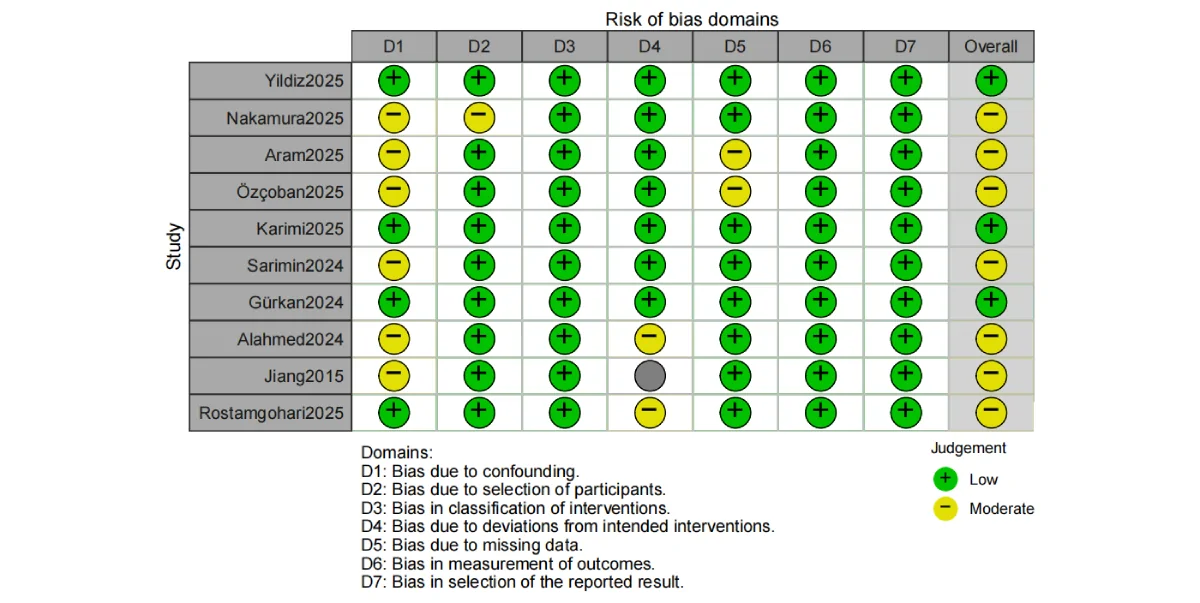
Visualization of the risk of bias of nonrandomized studies (n=10).

Among the quasi-experimental studies included, 7 had a moderate risk [80,82,96,106,108,111,112], and 3 had a low risk [86,88,98]. In addition, 6 of the included studies did not report or use matching, stratification, or statistical adjustment to balance baseline differences between groups, resulting in a moderate risk of bias [80,82,96,106,108,112]. Regarding bias due to selection of participants, 2 studies were rated as having a moderate risk of bias due to their small sample sizes and insufficient representativeness [106,125]. Regarding selection bias, 2 studies were assessed as having a moderate risk of bias because the study sample did not represent the expected characteristics of the target population [80,111]. Regarding attrition bias, 5 studies had high participant dropout rates, indicating a moderate risk of bias [82,108,125-127]. All studies had a low risk of bias in terms of classification of interventions, outcome measurement, and selective reporting (Figure 3 [80,82,86,88,96,98,106,108,111,112]).

### Certainty of Evidence

This systematic review applied the GRADE framework to assess the certainty of evidence for the effects of DHIs on breastfeeding outcomes. The certainty of evidence was moderate for EBF at <3 months and 3-6 months, downgraded by 1 level because of serious inconsistency (*I*^2^=84% and 75%, respectively). For EBF at ≥6 months, the certainty of evidence was low, downgraded for serious inconsistency (*I*^2^=86% and a wide 95% PI) and potential small-study effects identified by the Egger test (*P*=.006), although the pooled effect remained robust after trim-and-fill correction. The certainty of evidence for breastfeeding self-efficacy was moderate, downgraded because of serious inconsistency (*I*^2^=95% and a 95% PI crossing the null value). The certainty of evidence for breastfeeding knowledge was low, downgraded for serious inconsistency (*I*^2^=98% and a 95% PI crossing the null value) and serious imprecision due to the small number of studies (k=6), limited sample size, and an imprecise CI (Multimedia Appendix 5).

### Findings of Meta-Analysis

#### EBF Rates

**Figure 4:**
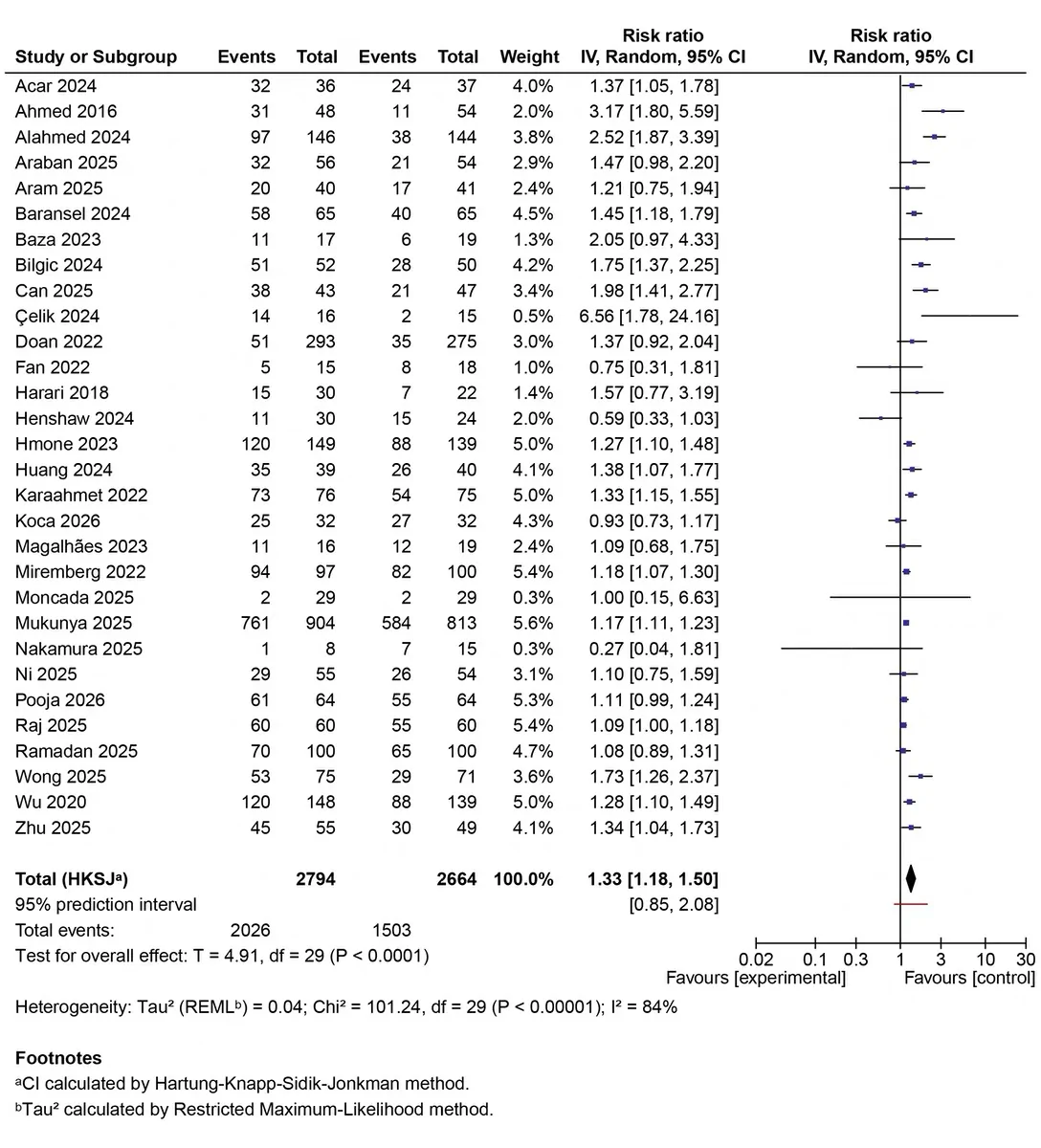
Forest plot of risk ratios (random-effects model) for the effect of digital health interventions on exclusive breastfeeding rates at <3 months, with 95% CIs, 95% prediction interval, and the overall pooled estimate. HKSJ: Hartung-Knapp-Sidik-Jonkman; IV: inverse variance; REML: restricted maximum likelihood.

**Figure 5:**
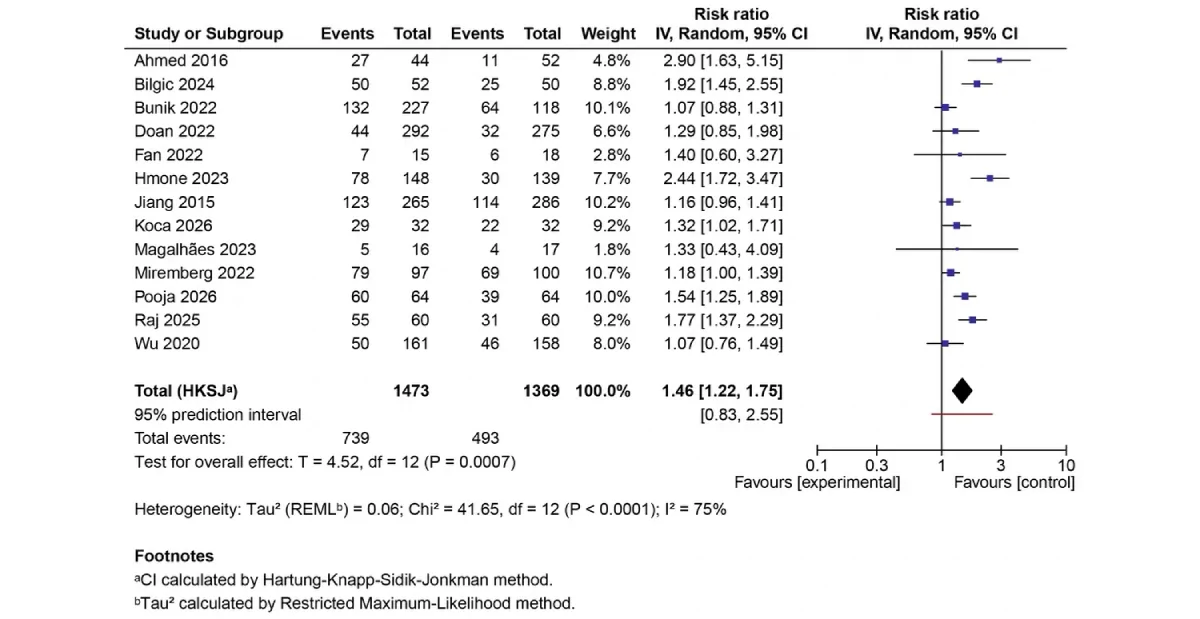
Forest plot of risk ratios (random-effects model) for the effect of digital health interventions on exclusive breastfeeding rates at 3-6 months, with 95% CIs, 95% prediction interval, and the overall pooled estimate. HKSJ: Hartung-Knapp-Sidik-Jonkman; IV: inverse variance; REML: restricted maximum likelihood.

**Figure 6:**
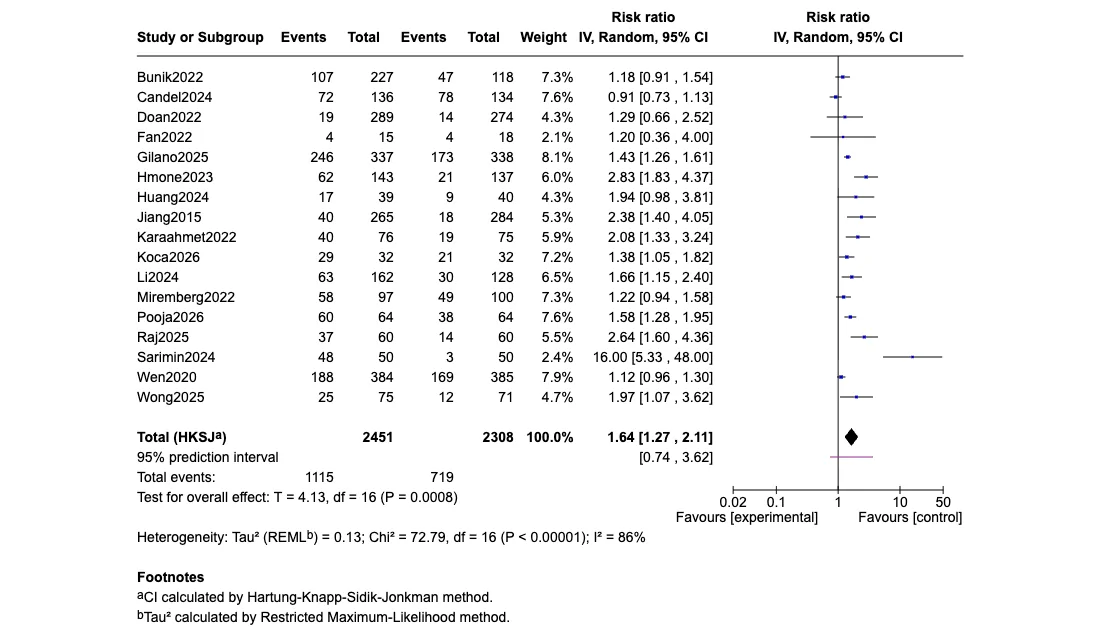
Forest plot of risk ratios (random-effects model) for the effect of digital health interventions on exclusive breastfeeding rates at ≥6 months, with 95% CIs, 95% prediction interval, and the overall pooled estimate. HKSJ: Hartung-Knapp-Sidik-Jonkman; IV: inverse variance; REML: restricted maximum likelihood.

A total of 30 studies reported EBF rates at <3 months [34,56,57,78-84,87,89-95,97,99,100,102,104-106,109,110,114,119,120], 13 studies reported rates at 3-6 months [56,57,79,84,85,89-91,96,99,100,102,109], and 17 studies reported rates at ≥6 months [42,43,57,85,89-91,94,96,97,99,102,109,112,117-119]. Participants who used DHIs had higher EBF rates than the control group at all time points (<3 months: RR 1.33, 95% CI 1.18-1.50; *I*^2^=84%; *P*<.001; 3-6 months: RR 1.46, 95% CI 1.22-1.75; *P*<.001; *I*^2^=75%; ≥6 months: RR 1.64, 95% CI 1.27-2.11; *P*<.001; *I*^2^=86%). The 95% PI for the point estimates (<3 months: 0.85-2.08; 3-6 months: 0.83-2.55; ≥6 months: 0.74-3.62) suggests that the true effect of future similar studies in different populations or settings may vary considerably. Although the pooled effect size results indicate that DHIs produces a favorable average effect, the intervention may have a negligible or even marginally negative impact on some patients or subgroups, which is consistent with the observed significant heterogeneity (<3 months: *P*<.001; τ^2^=0.04; *I*^2^*=*84%; Figure 4 [34,56,57,78-84,87,89-95,97,99,100,102,104-106,109,110,114,119,120]; 3-6 months: *P*<.001; τ^2^=0.06; *I*^2^=75%; Figure 5 [56,57,79,84,85,89-91,96,99,100,102,109]; ≥6 months: *P*<.001; τ^2^=0.13; *I*^2^=86%; Figure 6 [42,43,57,85,89-91,94,96,97,99, 102,109,112,117-119]).

#### Breastfeeding Self-Efficacy

**Figure 7:**
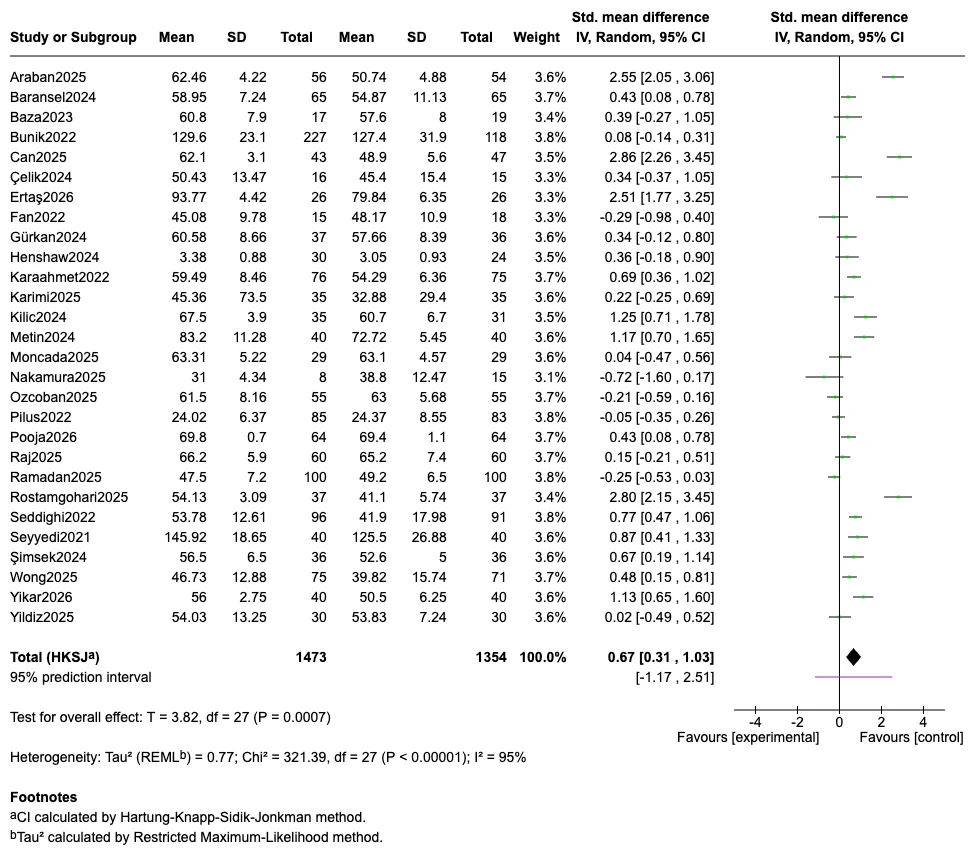
Forest plot of standardized mean differences (random-effects model) for the effect of digital health interventions on breastfeeding self-efficacy, with 95% CIs, 95% prediction interval, and the overall pooled estimate. HKSJ: Hartung-Knapp-Sidik-Jonkman; IV: inverse variance; REML: restricted maximum likelihood; SMD: standardized mean difference.

A total of 28 studies reported on breastfeeding self-efficacy [34,35,49,81,83,85-88,90,91,93,97,98,101,103,104,106,108-111,113-115,119,121,122]. Participants who used DHIs had higher breastfeeding self-efficacy scores than the control group (SMD 0.67, 95% CI 0.31-1.03; *P*<.001). The 95% PI (–1.17 to 2.51) indicates that while the average effect of DHIs was a positive improvement, some participants may have shown no improvement or even worse outcomes after receiving the DHIs intervention, while others may have experienced greater improvement. This significant variability in individual responses is consistent with the high heterogeneity observed in the study (*P*<.001; τ^2^=0.77; *I*^2^=95%; Figure 7 [34,35,49,81,83,85-88,90,91,93,97, 98,101,103,104,106,108-111,113-115,119,121,122]).

#### Breastfeeding Knowledge

**Figure 8:**
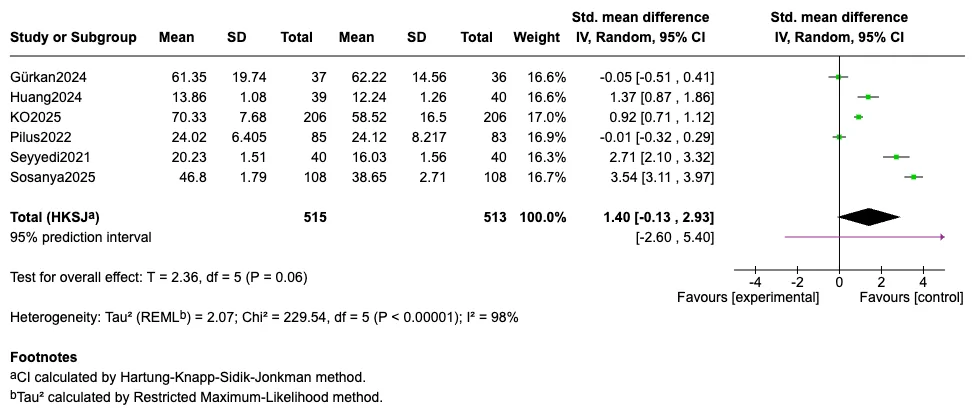
Forest plot of standardized mean differences (random-effects model) for the effect of digital health interventions on breastfeeding knowledge, with 95% CIs, 95% prediction interval, and the overall pooled estimate. HKSJ: Hartung-Knapp-Sidik-Jonkman; IV: inverse variance; REML: restricted maximum likelihood; SMD: standardized mean difference.

A total of 6 studies reported on breastfeeding knowledge [86,94,103,107,115,116]. The results showed that DHIs had no significant effect on improving breastfeeding knowledge (SMD 1.40, 95% CI –0.13 to 2.93; *P*=.06). The 95% PI was further estimated at (–2.60 to 5.40), and significant statistical heterogeneity was observed among the included trials (*P*<.001; τ^2^=2.07; *I*^2^*=*98%; Figure 8 [86,94,103,107,115,116]).

### Subgroup Analyses

#### Type of Intervention

##### EBF Rates

A subgroup analysis was conducted based on DHIs type. eHealth (<3 months: RR 1.56, 95% CI 1.12-2.17; *P*=.01; *I*^2^=90%; 3-6 months: RR 1.70, 95% CI 1.07-2.69; *P*=.04; *I*^2^=66%; ≥6 months: RR 1.59, 95% CI 1.25-2.02; *P*<.001; *I*^2^=0%), mHealth (<3 months: RR 1.21, 95% CI 1.13-1.29; *P*<.001; *I*^2^=29%; 3-6 months: RR 1.34, 95% CI 1.08-1.67; *P*=.01; *I*^2^=75%; ≥6 months: RR 1.68, 95% CI 1.16-2.42; *P*=.01; *I*^2^=91%), and digital devices (<3 months: RR 1.34, 95% CI 1.04-1.73; *P*=.03) increased EBF rates across all three time periods. No statistically significant differences were observed among subgroups (<3 months: *χ*^2^_2_=3.27; *P*=.19; 3-6 months: *χ*^2^_1_=1.83; *P*=.18; ≥6 months: *χ*^2^_1_=0.09; *P*=.77). Notably, heterogeneity for eHealth gradually decreased over time, suggesting that long-term effects (≥6 months) may be more stable, whereas mHealth may have a greater short-term (<3 months) effect (Multimedia Appendix 6).

##### Breastfeeding Self-Efficacy

A subgroup analysis was conducted based on DHI type. mHealth (SMD 0.58, 95% CI 0.07-1.10; *P*=.03; *I*^2^=93%) was found to improve participants’ breastfeeding self-efficacy, although the studies exhibited high heterogeneity. In contrast, eHealth (SMD 0.47, 95% CI –0.04 to 0.98; *P*=.07; *I*^2^=93%) and digital devices (SMD 1.63, 95% CI –0.39 to 3.65; *P*=.08; *I*^2^=94%) did not demonstrate statistically significant effects. Tests for differences between subgroups showed that the interventions did not reach statistical significance (*χ*^2^_2_=2.97; *P*=.23; Multimedia Appendix 6).

#### Period of Intervention

##### EBF Rates

Subgroup analyses were conducted based on the DHIs stage. Prenatal (<3 months: RR 1.42, 95% CI 1.25-1.63; *P*<.001; *I*^2^=79%; 3-6 months: RR 1.42, 95% CI 1.07-1.89; *P*=.02; *I*^2^=81%; ≥6 months: RR 1.68, 95% CI 1.16-2.44; *P*=.01; *I*^2^=91%) and postpartum (3-6 months: RR 1.52, 95% CI 1.10-2.11; *P*=.02; *I*^2^=68%; ≥6 months: RR 1.62, 95% CI 1.07-2.46; *P*=.03; *I*^2^=64%), whereas interventions during the postpartum period <3 months (RR 1.21, 95% CI 0.97-1.50; *P*=.08; *I*^2^=80%) did not reach statistical significance. Differences among subgroups were not statistically significant (<3 months: *χ*^2^_1_=1.94; *P*=.16; 3-6 months: *χ*^2^_1_=0.16; *P*=.69; ≥6 months: *χ*^2^_1_=0.03; *P*=.87; Multimedia Appendix 6).

##### Breastfeeding Self-Efficacy

Subgroup analyses were conducted based on the DHIs stage. Interventions during the prenatal period (SMD 0.70, 95% CI 0.18-1.22; *P*=.01; *I*^2^=96%) and the postnatal period (SMD 0.63, 95% CI 0.06-1.19; *P*=.03; *I*^2^=92%) were both statistically significant. However, both groups exhibited extremely high heterogeneity among studies. There was no statistically significant difference between the groups (*χ*^2^_2_=0.04; *P*=.84; Multimedia Appendix 6).

##### Breastfeeding Knowledge

A subgroup analysis was conducted based on DHIs stages. Interventions during the prenatal (SMD 0.66, 95% CI –8.10 to 9.42; *P*=.51; *I*^2^=95%) and postnatal (SMD 1.77, 95% CI –0.83 to 4.38; *P*=.11; *I*^2^=99%) interventions had no statistically significant effect on breastfeeding knowledge, and there was very high heterogeneity. Differences between groups did not reach statistical significance (*χ*^2^_1_=1.08; *P*=.30; Multimedia Appendix 6).

#### Duration of Intervention

##### EBF Rates

A subgroup analysis was conducted based on intervention duration (<3 months vs ≥3 months). An intervention lasting <3 months had a significant effect only on short-term EBF rates (RR 1.41, 95% CI 1.09-1.83; *P*=.01; *I*^2^=93%); it had no statistically significant effect on midterm (3-6 months) or long-term (≥6 months) EBF rates. Due to high heterogeneity among studies and the extremely small number of studies in the ≥6-month group (n=2), with CIs spanning a wide range, caution is warranted when interpreting the subgroup analysis. For interventions lasting ≥3 months (<3 months: RR 1.28, 95% CI 1.15-1.42; *P*<.001; *I*^2^=55%; 3-6 months: RR 1.37, 95% CI 1.13-1.66; *P*=.004; *I*^2^=74%; ≥6 months: RR 1.69, 95% CI 1.35-2.11; *P*<.001; *I*^2^=82%) all increased the rates of EBF during their respective time periods. No significant statistical differences were observed among subgroups (<3 months: *χ*^2^_2_=0.60; *P*=.44; 3-6 months: *χ*^2^_1_=3.53; *P*=.06; ≥6 months: *χ*^2^_1_=0.76; *P*=.38), with the test for differences between groups in the 3-6-month period showing a marginally significant effect, indicating that the duration of dry interventions may have a potential impact on the overall heterogeneity of the meta-analysis, although it did not reach formal statistical significance, and the point estimates still favored shorter intervention durations (RR 1.98, 95% CI 0.93-4.22; *P*=.06; *I*^2^=30%; Multimedia Appendix 6).

##### Breastfeeding Self-Efficacy

A subgroup analysis was conducted based on intervention duration (<3 months vs ≥3 months). Interventions lasting <3 months (SMD 0.64, 95% CI 0.19-1.08; *P*=.008; *I*^2^=94%) showed a statistically significant positive effect on breastfeeding self-efficacy, whereas interventions lasting ≥3 months (SMD 0.73, 95% CI –0.01 to 1.48; *P*=.05; *I*^2^=96%) demonstrated a marginally significant effect, with the CI straddling the null line, and thus did not reach statistical significance. The subgroup analysis revealed no statistically significant differences among subgroups (*χ*^2^_1_=0.06; *P*=.81), but the test for differences among subgroups indicated a marginally significant effect, suggesting that intervention duration may have a potential impact on the overall effect of the meta-analysis (Multimedia Appendix 6).

##### Breastfeeding Knowledge

A subgroup analysis was conducted based on intervention duration (<3 months vs ≥3 months). The subgroup analysis revealed a statistically significant difference between subgroups (*χ*^2^_1_=12.51; *P*<.001). For interventions lasting <3 months (SMD –0.02, 95% CI –0.23 to 0.19; *P*=.38; *I*^2^=0%), whereas interventions lasting ≥3 months (SMD 2.12, 95% CI 0.19-4.05; *P*=.04; *I*^2^=97%) showed statistical significance, suggesting that long-term interventions may be more effective than short-term ones. However, there was very high heterogeneity within the subgroup, limiting the stability of the effect estimates; therefore, the results should be interpreted with caution (Multimedia Appendix 6).

#### Frequency of Intervention

##### EBF Rates

A subgroup analysis was conducted based on intervention frequency (<3 times/week vs ≥3 times/week). Interventions <3 times/week showed greater effects on short-term (<3 months) EBF rates (RR 1.32, 95% CI 1.03-1.68; *P*=.04; *I*^2^=61%) and interventions ≥3 times/week on <3-month EBF rates (RR 1.29, 95% CI 1.10-1.51; *P*=.007; *I*^2^=53%). No statistically significant effects were observed in the subgroups for intermediate-term (3-6 months) and long-term (≥6 months) intervention frequencies. No significant statistical differences were observed between subgroups (<3 months: *χ*^2^_1_=0.04; *P*=.84; 3-6 months: *χ*^2^_1_=0.01; *P*=.93; ≥6 months: *χ*^2^_1_=1.16; *P*=.28; Multimedia Appendix 6).

##### Breastfeeding Self-Efficacy

Subgroup analyses were conducted based on intervention frequency (<3 times/week vs ≥3 times/week). For interventions <3 times/week (SMD 0.97, 95% CI –0.90 to 2.83; *P*=.20; *I*^2^=97%) and interventions ≥3 times/week (SMD 0.35, 95% CI –3.76 to 4.46; *P*=.48; *I*^2^=70%) had no statistically significant effect on breastfeeding self-efficacy, and high heterogeneity was observed. The subgroup analysis revealed no statistically significant differences among subgroups (*χ*^2^_1_=0.85; *P*=.36; Multimedia Appendix 6).

#### National Income Level

##### EBF Rate

An exploratory subgroup analysis was conducted based on country (developing vs developed countries). In developing countries, DHIs can increase the EBF rate across intervention durations of <3 months (RR 1.32, 95% CI 1.19-1.47; *P*<.001; *I*^2^=82%), 3-6 months (RR 1.45, 95% CI 1.21-1.75; *P*=.001; *I*^2^=72%) and ≥6 months (RR 1.83, 95% CI 1.50-2.23; *P*<.001; *I*^2^=70%), while no statistically significant effects were observed in developed countries. The subgroup analysis showed no statistically significant differences between subgroups (<3 months: *χ*^2^_1_=0.01; *P*=.93; 3-6 months: *χ*^2^_1_=0.07; *P*=.79; ≥6 months: *χ*^2^_1_=0.01; *P*=.91). The uneven distribution and high heterogeneity across subgroups may reduce the statistical power of subgroup comparisons; therefore, these subgroup results should be interpreted with caution (Multimedia Appendix 6).

##### Breastfeeding Self-Efficacy

An exploratory subgroup analysis was conducted based on country (developing vs developed countries). There were statistically significant differences between subgroups (*χ*^2^_1_=8.26; *P*=.004); DHIs were higher in developing countries (SMD 0.77, 95% CI 0.37-1.18; *P*<.001; *I*^2^=95%) compared to developed countries (SMD 0.11, 95% CI –0.29 to 0.51; *P*=.46; *I*^2^=0%). However, there was very high heterogeneity among subgroups, and the stability of the effect estimates was limited; therefore, the results should be interpreted with caution (Multimedia Appendix 6). All subgroup comparisons constitute post hoc exploratory analyses.

#### Population Characteristics

##### EBF Rates

The intervention was more effective among mothers of preterm infants (RR 1.08, 95% CI 1.03-1.13; *P*=.03; *I*^2^=0%) compared to mothers of full-term infants (RR 1.35, 95% CI 1.19-1.53; *P*<.001; *I*^2^=85%). There was a statistically significant difference between subgroups (*χ*^2^_1_=13.24; *P*<.001), suggesting that gestational age may have a significant impact on the overall heterogeneity of the meta-analysis. For adult mothers, the intervention increased the rate of EBF among infants under 3 months of age (RR 1.30, 95% CI 1.16-1.45; *P*<.001; *I*^2^=82%), whereas no significant effect was observed for adolescent mothers. The subgroup analysis revealed no statistically significant differences between subgroups (*χ*^2^_1_=2.17; *P*=.14). Regarding mode of delivery, DHIs significantly increased the EBF rates among mothers who had a cesarean section (<3 months: RR 1.34, 95% CI 1.21-1.48; *P*=.02; *I*^2^=0%) but had no statistically significant effect on midterm or long-term EBF rates. At the same time, DHIs had no statistically significant effect on the EBF rates at any of the three time points among mothers who delivered vaginally. Differences between groups did not reach statistical significance (≤3 months: *χ*^2^_1_=0.54; *P*=.46; 3-6 months: *χ*^2^_1_=0.18; *P*=.67; ≥6 months: *χ*^2^_1_=1.44; *P*=.23; Multimedia Appendix 6).

##### Breastfeeding Self-Efficacy

DHIs were associated with increased breastfeeding self-efficacy among full-term mothers (SMD 0.63, 95% CI 0.29-0.97; *P*<.001; *I*^2^=93%), adult mothers (SMD 0.60, 95% CI 0.26-0.95; *P*=.001; *I*^2^=94%), singleton mothers (SMD 0.57, 95% CI 0.18-0.96; *P*=.007; *I*^2^=91%), and cesarean section mothers (SMD 0.97, 95% CI 0.22-1.73; *P*=.03; *I*^2^=50%), whereas there was no significant effect on breastfeeding self-efficacy among mothers of preterm infants, adolescent mothers, mothers of multiple births, and mothers who delivered vaginally. Notably, a statistically significant difference was observed between subgroups based on number of fetuses (*χ*^2^_1_=6.78; *P*=.009), which may have a potential impact on the overall heterogeneity of the intervention (Multimedia Appendix 6).

### Small-Study Effects and Sensitivity Analysis

This meta-analysis used the Egger regression test to assess funnel plot symmetry and investigate potential small-study effects. The results showed that the funnel plots for EBF rates at <3 months and 3-6 months, breastfeeding self-efficacy, and breastfeeding knowledge were symmetrical, whereas the funnel plot for EBF rates at ≥6 months indicated asymmetry, suggesting a potential small-study effect. This may stem from publication bias, genuine heterogeneity, or methodological discrepancies across studies. The trim-and-fill method was used to assess potential small-study effects. Results showed that the adjusted effect size remained significant (RR 1.70, 95% CI 1.29-2.24; *P*<.001; *I*^2^=93%) even without adding missing studies (Multimedia Appendix 7). Notably, heterogeneity remained high after adjustment, indicating substantial between-study variability; therefore, the results should be interpreted with caution. The sensitivity analysis, conducted by sequentially removing each study, confirmed that no single study had an excessive influence on the overall pooled estimate of breastfeeding outcomes, demonstrating the robustness of the findings (Multimedia Appendix 8).

## Discussion

### Principal Findings

The meta-analysis confirmed that DHIs have a statistically reliable average effect on EBF rates and breastfeeding self-efficacy, but no statistically significant effect was observed for breastfeeding knowledge. Notably, these pooled estimates represent average effects across highly diverse study contexts. A wide 95% PI, however, indicates substantial real-world variability in individual DHI responses, restricting broad clinical deployment. Subgroup analyses also reflected this inconsistency, although intervention duration, country, gestational age, and number of fetuses provided some explanatory power; heterogeneity remained high in some subgroups and sample distribution was uneven, necessitating further research to elucidate the underlying causes of heterogeneity. These findings underscore that the potential of DHIs to improve breastfeeding outcomes still requires further validation.

Research has found that DHIs can increase EBF rates and breastfeeding self-efficacy, which is consistent with previous conclusions regarding the role of digital or remote support in promoting breastfeeding outcomes. These benefits may result from personalized guidance, timely feedback, and emotional support provided by DHIs [128]. Existing evidence suggests that prenatal and postpartum breastfeeding education, particularly for first-time mothers, is critical in improving early breastfeeding practices such as latching technique and feeding management [5,129]. By overcoming temporal and geographic barriers, DHIs extend traditional support through online consultation [56,130,131], multimedia education [132], and interactive feedback [133], enabling mothers to identify and correct breastfeeding problems promptly. In addition, peer support and online counseling components may reduce postpartum anxiety and strengthen maternal confidence [134]. Emerging technologies, such as VR-based demonstrations, may further facilitate skill acquisition [25,122]. Collectively, these integrated functions may contribute to both improved EBF practices and enhanced maternal breastfeeding self-efficacy.

It is important to note that the 95% PI for the effects of DHIs on both EBF rates and breastfeeding self-efficacy are wide and cross the line of no effect. This suggests substantial inconsistency in the expected effects of DHIs in future real-world settings, indicating that while the average effect is positive, the intervention may yield null or even negative outcomes in some contexts or subgroups. Such variability likely reflects true differences across populations, intervention designs, and implementation conditions, including differences in user engagement, intervention intensity, duration, and technological complexity. In addition, variations in outcome measurement tools may further contribute to inconsistencies across studies. Therefore, these findings highlight that the pooled estimates should be interpreted as average effects across highly diverse settings, rather than uniform effects applicable to all populations.

The impact of DHIs on breastfeeding knowledge remains uncertain, which differs from earlier systematic reviews that reported improvements in breastfeeding knowledge [135,136]. However, knowledge acquisition does not necessarily translate into behavioral change, as effective breastfeeding education requires sustained engagement, repeated reinforcement, and appropriate intervention intensity [137]. Excessive or poorly tailored information may lead to information overload and reduced maternal engagement [127,138,139], while factors such as baseline knowledge, educational level, prior breastfeeding experience, and lack of individualized intervention design may further weaken the effectiveness of standardized programs [139-141].

The 95% PI for breastfeeding knowledge indicates substantial variability in intervention effects across populations and settings, suggesting pronounced individual response differences. This uncertainty is further reflected by methodological limitations, including a small number of included studies, lack of standardized outcome measurement tools, and inadequate adjustment for key confounders, which may introduce bias and affect the precision of pooled estimates. Although the evidence certainty was rated as moderate and the *P* value approached statistical significance (*P*=.06), this does not provide conclusive evidence of ineffectiveness. Overall, these findings suggest that while the average effect may be limited, DHIs may still be beneficial in specific contexts or subgroups that are not captured by aggregate analyses, highlighting the need for larger, high-quality studies with standardized designs.

### Subgroup Analyses

#### EBF Rates

Given substantial heterogeneity across studies, subgroup analyses were conducted according to predefined intervention characteristics (type, phase, frequency, and duration). Overall, all intervention types were associated with higher EBF rates compared with controls, but no significant between-group differences were observed. mHealth interventions appeared to produce greater short-term benefits, likely because of their accessibility, ease of use, and frequent reminders [52,142], whereas more comprehensive eHealth interventions, such as web-based platforms and telemedicine, may provide more sustained effects through personalized content, interactive support, and continuous breastfeeding guidance [98,143]. However, evidence regarding digital device-based interventions remains limited because only 1 eligible study was included and requires further verification. Both prenatal and postnatal interventions can increase EBF rates. Effect sizes tended to be larger and more stable for 3-6 months and ≥6 months compared with <3 months, although subgroup differences were not statistically significant. Substantial within-subgroup heterogeneity persisted, indicating that intervention duration alone does not fully explain between-study variability. This may reflect the practical challenges encountered during the early postpartum period, when mothers often require timely, face-to-face support for breastfeeding difficulties that digital interventions cannot fully replace [110,144,145]. Subgroup analyses showed that neither intervention frequency nor duration was a significant effect modifier for EBF rates, as no statistically significant between-subgroup differences were observed. For frequency, low- and high-frequency interventions both produced significant short-term effects with comparable pooled estimates, indicating no clear dose-response gradient [146,147]. For duration, interventions lasting ≥3 months generally showed more stable effects across follow-up periods, while shorter interventions mainly influenced early outcomes [146,148]; however, the subgroup difference was not significant. Overall, moderate to high within-subgroup heterogeneity suggests that variability is likely driven by factors beyond frequency and duration.

Exploratory subgroup analyses by national income level showed consistent effect directions across postpartum periods, with no significant between-group differences, suggesting limited modification by income level. Effect estimates tended to be larger in developing countries, whereas high-income settings showed nonsignificant or less precise effects with greater heterogeneity. These differences should not be interpreted solely in terms of economic development. They may also reflect variations in health system capacity (eg, availability and continuity of lactation support, standardized counseling, and follow-up) [12,19,149,150], regulation of formula marketing, and sociocultural contexts including breastfeeding norms, family support, and maternity leave and workplace policies [151-154]. Similarly, mothers of preterm infants, adult mothers, and women undergoing cesarean delivery appeared to derive greater benefits from DHIs, likely reflecting their increased need for breastfeeding guidance and support. However, these subgroup analyses were based on relatively few studies, and most between-group differences were not statistically significant. Given the exploratory nature and substantial heterogeneity, these findings should be interpreted as hypothesis-generating.

#### Breastfeeding Self-Efficacy

Overall, DHIs improved breastfeeding self-efficacy, with significant effects primarily driven by mHealth. This may relate to their low access threshold, reminder functions, and structured feedback, which facilitate sustained maternal engagement [50,95,130,155,156]. Considerable between-study heterogeneity was observed, likely reflecting differences in intervention complexity, intensity, and user engagement. No clear superiority was identified across eHealth and digital devices; subgroup differences were nonsignificant. Intervention frequency did not significantly moderate DHI effects on breastfeeding self-efficacy, with no evidence of a dose-response relationship [147,157]. Both prenatal and postnatal DHIs showed overall beneficial effects, but with substantial heterogeneity and nonsignificant subgroup differences, indicating limited explanatory value of timing in the pooled moderation tests [158,159]. However, short-term and early postpartum-initiated interventions demonstrated clearer benefits, whereas longer-term or delayed interventions showed attenuated or unstable effects [160]. This suggests that timing of support may be more important than repetition intensity in influencing breastfeeding self-efficacy. Overall heterogeneity remained high, and most subgroup effects were nonsignificant, indicating that observed differences should be interpreted cautiously and may be driven by unmeasured contextual factors rather than true effect modification.

An exploratory subgroup analysis by national income level showed significant effects in developing countries but not in developed settings, with significant between-group differences. However, substantial within-group heterogeneity in developing countries limits the stability of pooled estimates. DHIs may have greater impact in resource-limited settings due to weaker baseline support and greater unmet needs [149,150], while effects appear attenuated in well-resourced contexts [153,154]. Across maternal and infant characteristics, benefits were observed in several subgroups, including term infants, singletons, adult mothers, and cesarean deliveries, whereas others showed inconsistent or null effects, with fetal multiplicity emerging as the only significant moderator. These findings suggest potential effect modification by clinical and caregiving burden. Overall, most subgroup differences were nonsignificant, and findings remain exploratory given high heterogeneity and limited statistical power.

#### Breastfeeding Knowledge

A subgroup analysis by intervention phase showed no significant effects for either postnatal interventions or prenatal interventions, with no significant between-group difference, suggesting limited explanatory value of intervention timing. In contrast, intervention duration demonstrated a significant subgroup effect. Interventions lasting ≥3 months significantly improved knowledge, whereas shorter interventions (<3 months) showed no effect, with a significant between-group difference. This pattern is consistent with a cumulative learning effect, where longer exposure allows repeated reinforcement and consolidation of breastfeeding knowledge, whereas short-term interventions may be insufficient to induce measurable knowledge change [19,146].

### Strengths and Limitations

This meta-analysis followed rigorous methodological guidelines for systematic reviews, with comprehensive searches and sensitivity analyses supporting the robustness of findings. Furthermore, this review features broad inclusivity regarding study populations and interventions. Compared with previous reviews limited to telephone, SMS text messages, or mobile apps, our study included emerging DHIs, such as wearable devices and AI-based interventions. We also expanded the population scope to include women across pregnancy and postpartum stages and conducted comprehensive subgroup analyses to identify key technical parameters of the interventions and elucidated the varying effects of DHIs across countries with different levels of economic development and among different maternal populations. In addition, the inclusion of recent trials and GRADE assessment strengthened the reliability of evidence. These findings provide insights for optimizing digital breastfeeding support and suggest that DHIs may complement traditional care by improving accessibility and continuity of support.

However, this systematic review also has limitations. First, substantial heterogeneity remained in some outcomes despite predefined subgroup analyses, likely due to differences in intervention characteristics, user engagement, measurement tools, and participant populations. Second, the limited number of studies and uneven subgroup distribution reduced statistical power and estimate precision. Third, the inclusion of quasi-experimental studies may have introduced selection, performance, and measurement biases, while variations in outcome assessment tools and restriction to English-language publications may further affect comparability and increase bias risk. Moreover, the overall evidence certainty was low to moderate, and findings should therefore be interpreted cautiously [161]. Evidence for emerging technologies, such as AI-based chatbots and machine learning interventions, remains limited. Future well-designed, adequately powered RCTs with standardized outcomes and subgroup analyses are needed to clarify heterogeneity and evaluate effectiveness, acceptability, cost-effectiveness, and dose-response relationships.

### Conclusions

This study comprehensively assessed the impact of DHIs on breastfeeding outcomes across multiple dimensions (type, duration, stage, frequency, country, and population characteristics) and outcomes (EBF rate, breastfeeding self-efficacy, and breastfeeding knowledge). Studies suggest that digital interventions may effectively improve EBF rates and breastfeeding self-efficacy, yet no statistically significant evidence currently supports their benefits for breastfeeding knowledge. Exploratory subgroup analyses revealed comparatively stronger intervention benefits in several specific subgroups, namely mHealth interventions, programs lasting a minimum of three months, studies carried out in developing countries, adult maternal samples, and women who underwent cesarean delivery. Our pooled analysis indicated that average pooled effects favor DHIs, but significant between-study heterogeneity and methodological differences among included studies may further limit the generalizability of pooled estimates, and applicability across settings remains uncertain. This study only provides preliminary exploratory evidence for the implementation of digital interventions in breastfeeding support. Larger, rigorously designed RCTs are warranted in future research to verify the efficacy of digital health technologies within real-world contexts and generate evidence to inform optimal intervention strategies tailored to diverse populations.

## Appendix

Multimedia Appendix 1 PRISMA 2020 checklist.

Multimedia Appendix 2 PRISMA-S checklist.

Multimedia Appendix 3 Search strategy.

Multimedia Appendix 4 Study characteristics.

Multimedia Appendix 5 Certainty of evidence.

Multimedia Appendix 6 Forest plot of subgroup analysis.

Multimedia Appendix 7 Funnel plot of the primary outcomes.

Multimedia Appendix 8 Sensitivity analysis.

## Abbreviations

DHI: digital health intervention
EBF: exclusive breastfeeding
GRADE: Grading of Recommendations, Assessment, Development and Evaluation
mHealth: mobile health
PI: prediction interval
PRISMA: Preferred Reporting Items for Systematic Reviews and Meta-Analyses
PRISMA-S: Preferred Reporting Items for Systematic Reviews and Meta-Analyses literature search extension
PROSPERO: International Prospective Register of Systematic Reviews
RCT: randomized controlled trial
RoB2: Cochrane Risk-of-Bias Tool version 2
ROBINS-I: Risk of Bias in Nonrandomized Studies of Interventions
RR: relative risk
SMD: standardized mean difference
VR: virtual reality
WHO: World Health Organization

## Data Availability

The datasets generated or analyzed during this study are available from the corresponding author on reasonable request.
