# Supplementary material for: Effects of Digital Health Interventions on Breastfeeding Rates, Self-Efficacy, and Knowledge: Systematic Review and Meta-Analysis
Source: J Med Internet Res. 2026 Aug 12;28:e92184. doi: 10.2196/92184 (PMC13519512; doi:10.2196/92184)
Supplement: Supplementary file 3 [file jmir_v28i1e92184_app3.docx]

**Multimedia Appendix 2**

Table S1. Literature search strategy.

**Database: CINAHL**

|  | **Searches** | **Results** |
| --- | --- | --- |
| **S1** | MH "Breast Feeding" OR TI (Breastfeeding OR Breast Feedings OR Breast Fed OR Breastfed OR Chestfeedings OR Breastfeeding OR Breast Feeding, Exclusive OR Exclusive Breast Feeding OR Breastfeeding, Exclusive OR Exclusive Breastfeeding OR Wet Nursing OR Milk Sharing OR Sharing, Milk) OR AB (Breastfeeding OR Breast Feedings OR Breast Fed OR Breastfed OR Chestfeedings OR Breastfeeding OR Breast Feeding, Exclusive OR Exclusive Breast Feeding OR Breastfeeding, Exclusive OR Exclusive Breastfeeding OR Wet Nursing OR Milk Sharing OR Sharing, Milk) | 40158 |
| **S2** | MH "Artificial Intelligence" OR TI (Artificial Intelligence OR Intelligence, Artificial OR Computer Reasoning OR Reasoning, Computer OR AI OR Machine Intelligence OR Intelligence, Machine OR Computational Intelligence OR Intelligence, Computational) OR AB (Artificial Intelligence OR Intelligence, Artificial OR Computer Reasoning OR Reasoning, Computer OR AI OR Machine Intelligence OR Intelligence, Machine OR Computational Intelligence OR Intelligence, Computational) | 42281 |
| **S3** | MH "Artificial Intelligence, Generative" OR TI (Generative Artificial Intelligence or Artificial Intelligence, Generative or Chatbot or Chatbots or ChatGPTs or Chat GPT or ChatGPT or Chat-GPT) OR AB (Generative Artificial Intelligence or Artificial Intelligence, Generative or Chatbot or Chatbots or ChatGPTs or Chat GPT or ChatGPT or Chat-GPT) | 5662 |
| **S4** | (large language models or llm or nlp or natural language processing) OR TI (large language models or llm or nlp or natural language processing) OR AB (large language models or llm or nlp or natural language processing) | 8962 |
| **S5** | MH "Machine Learning" OR TI (Machine Learning or Transfer Learning or Learning, Transfer or Learning, Machine) OR AB (Machine Learning or Transfer Learning or Learning, Transfer or Learning, Machine) | 35361 |
| **S6** | MH "Deep Learning" OR TI (Deep Learning or Hierarchical Learning or Learning, Deep or Learning, Hierarchical) OR AB (Deep Learning or Hierarchical Learning or Learning, Deep or Learning, Hierarchical) | 12034 |
| **S7** | MH "Machine Learning Algorithms" OR TI (Algorithms, Machine Learning OR Machine-Learning Algorithms) OR AB (Algorithms, Machine Learning OR Machine-Learning Algorithms) | 5650 |
| **S8** | MH "Wearable Sensors" OR TI (Wearable Electronic Devices or Device, Wearable Electronic or Electronic Device, Wearable or Wearable Electronic Device or Wearable Devices or Device, Wearable or Wearable Device or Wearable Technology or Technology, Wearable or Wearable Technologies or Electronic Skin or Skin, Electronic or Wearable Computer or Computer, Wearable or Wearable Computers) OR AB (Wearable Electronic Devices or Device, Wearable Electronic or Electronic Device, Wearable or Wearable Electronic Device or Wearable Devices or Device, Wearable or Wearable Device or Wearable Technology or Technology, Wearable or Wearable Technologies or Electronic Skin or Skin, Electronic or Wearable Computer or Computer, Wearable or Wearable Computers) | 7962 |
| **S9** | MH "Telemedicine" OR TI (Telemedicine or Medicine, Virtual or Tele-Referrals or Tele Referral or Virtual Medicine or Tele-Referral or Tele-Intensive Care or Tele ICU or Tele Intensive Care or Tele-ICU or Telecare or Tele-Care or Tele Care or Health, Mobile or mHealth or Mobile Health or Telehealth or eHealth) OR AB (Telemedicine or Medicine, Virtual or Tele-Referrals or Tele Referral or Virtual Medicine or Tele-Referral or Tele-Intensive Care or Tele ICU or Tele Intensive Care or Tele-ICU or Telecare or Tele-Care or Tele Care or Health, Mobile or mHealth or Mobile Health or Telehealth or eHealth) | 41891 |
| **S10** | MH "Mobile Applications" OR TI (Mobile Applications or Mobile Application or Apps, Mobile or Mobile Apps or App, Mobile or Application, Mobile or Applications, Mobile or Mobile App or Smartphone Apps or Apps, Smartphone or App, Smartphone or Smartphone App or Portable Electronic Application or Portable Electronic Apps or Electronic App, Portable or App, Portable Electronic or Portable Electronic Applications or Application, Portable Electronic or Electronic Application, Portable or Portable Electronic App or Software Application, Portable or Application, Portable Software or Portable Software Applications or Software App, Portable or Portable Software Apps or App, Portable Software or Portable Software App or Portable Software Application) OR AB (Mobile Applications or Mobile Application or Apps, Mobile or Mobile Apps or App, Mobile or Application, Mobile or Applications, Mobile or Mobile App or Smartphone Apps or Apps, Smartphone or App, Smartphone or Smartphone App or Portable Electronic Application or Portable Electronic Apps or Electronic App, Portable or App, Portable Electronic or Portable Electronic Applications or Application, Portable Electronic or Electronic Application, Portable or Portable Electronic App or Software Application, Portable or Application, Portable Software or Portable Software Applications or Software App, Portable or Portable Software Apps or App, Portable Software or Portable Software App or Portable Software Application) | 21575 |
| **S11** | MH "Text Messaging" OR TI (Text Messaging OR Messaging, Text OR Texting OR Short Message Service OR Text Messages OR Messages, Text OR Message, Text OR Text Message) OR AB (Text Messaging OR Messaging, Text OR Texting OR Short Message Service OR Text Messages OR Messages, Text OR Message, Text OR Text Message) | 7996 |
| **S12** | MH "Virtual Reality" OR TI (Virtual Reality OR Reality, Virtual OR Virtual Reality, Educational OR Educational Virtual Realities OR Educational Virtual Reality OR Reality, Educational Virtual OR Virtual Realities, Educational OR Virtual Reality, Instructional OR Instructional Virtual Realities OR Instructional Virtual Reality OR Realities, Instructional Virtual OR Reality, Instructional Virtual OR Virtual Realities, Instructional OR digital technology OR new technology OR immersive multimedia OR virtual environment OR video game OR virtual game OR virtual simulation OR virtual patient OR virtual patients OR patient simulation OR patient simulations OR virtual reality simulation devices OR virtual reality simulation system OR VR simulation system OR VR simulator OR virtual reality simulator) OR AB (Virtual Reality OR Reality, Virtual OR Virtual Reality, Educational OR Educational Virtual Realities OR Educational Virtual Reality OR Reality, Educational Virtual OR Virtual Realities, Educational OR Virtual Reality, Instructional OR Instructional Virtual Realities OR Instructional Virtual Reality OR Realities, Instructional Virtual OR Reality, Instructional Virtual OR Virtual Realities, Instructional OR digital technology OR new technology OR immersive multimedia OR virtual environment OR video game OR virtual game OR virtual simulation OR virtual patient OR virtual patients OR patient simulation OR patient simulations OR virtual reality simulation devices OR virtual reality simulation system OR VR simulation system OR VR simulator OR virtual reality simulator) | 38466 |
| **S13** | MH "Internet-Based Intervention" OR TI (Internet-Based Intervention OR Internet Based Intervention OR Internet-Based Interventions OR Intervention, Internet-Based OR Interventions, Internet-Based OR Internet Intervention OR Internet Interventions OR Intervention, Internet OR Interventions, Internet OR Web-based Intervention OR Interventions, Web-based OR Intervention, Web-based OR Web based Intervention OR Web-based Interventions OR Online Intervention OR Intervention, Online OR Interventions, Online OR Online Interventions) OR AB (Internet-Based Intervention OR Internet Based Intervention OR Internet-Based Interventions OR Intervention, Internet-Based OR Interventions, Internet-Based OR Internet Intervention OR Internet Interventions OR Intervention, Internet OR Interventions, Internet OR Web-based Intervention OR Interventions, Web-based OR Intervention, Web-based OR Web based Intervention OR Web-based Interventions OR Online Intervention OR Intervention, Online OR Interventions, Online OR Online Interventions) | 8835 |
| **S14** | S13 OR S12 OR S11 OR S10 OR S9 OR S8 OR S7 OR S6 OR S5 OR S4 OR S3 OR S2 | 190055 |
| **S15** | MH "Randomized Controlled Trials" OR TI (Randomized Controlled Trial or Randomized Controlled Trials as Topic or Randomized Controlled Trials as Topic or Clinical Trials, Randomized or Trials, Randomized Clinical or Controlled Clinical Trials,Randomized or Random Allocation or Allocation, Random or Randomization) OR AB (Randomized Controlled Trial or Randomized Controlled Trials as Topic or Randomized Controlled Trials as Topic or Clinical Trials, Randomized or Trials, Randomized Clinical or Controlled Clinical Trials,Randomized or Random Allocation or Allocation, Random or Randomization) | 283213 |
| **S16** | MH "Nonrandomized Trials" OR TI (Non-Randomized Controlled Trials as Topic OR Non Randomized Controlled Trials as Topic OR Nonrandomized Controlled Trials as Topic OR Clinical Trials, Nonrandomized OR Clinical Trial, Nonrandomized OR Nonrandomized Clinical Trial OR Nonrandomized Clinical Trials OR Trial, Nonrandomized Clinical OR Trials, Nonrandomized Clinical OR Controlled Clinical Trials, Non-Randomized OR Controlled Clinical Trials, Non Randomized OR Quasi-Experimental Studies OR Quasi Experimental Studies OR Quasi-Experimental Study OR Studies, Quasi-Experimental OR Study, Quasi-Experimental OR Clinical Trials, Non-Randomized OR Clinical Trial, Non-Randomized OR Clinical Trials, Non Randomized OR Non-Randomized Clinical Trial OR Non-Randomized Clinical Trials OR Trial, Non-Randomized Clinical OR Trials, Non-Randomized Clinical OR Controlled Clinical Trials, Nonrandomized) OR AB (Non-Randomized Controlled Trials as Topic OR Non Randomized Controlled Trials as Topic OR Nonrandomized Controlled Trials as Topic OR Clinical Trials, Nonrandomized OR Clinical Trial, Nonrandomized OR Nonrandomized Clinical Trial OR Nonrandomized Clinical Trials OR Trial, Nonrandomized Clinical OR Trials, Nonrandomized Clinical OR Controlled Clinical Trials, Non-Randomized OR Controlled Clinical Trials, Non Randomized OR Quasi-Experimental Studies OR Quasi Experimental Studies OR Quasi-Experimental Study OR Studies, Quasi-Experimental OR Study, Quasi-Experimental OR Clinical Trials, Non-Randomized OR Clinical Trial, Non-Randomized OR Clinical Trials, Non Randomized OR Non-Randomized Clinical Trial OR Non-Randomized Clinical Trials OR Trial, Non-Randomized Clinical OR Trials, Non-Randomized Clinical OR Controlled Clinical Trials, Nonrandomized) | 14557 |
| **S17** | S16 OR S15 | 295208 |
| **S18** | S18 AND S14 AND S1 | 138 |
| **S19** | S18 AND S14 AND S1  start date: 2025-10-01  end date: 2026-04-30 | 8 |

**Database: Cochrane Library**

|  | **Searches** | **Results** |
| --- | --- | --- |
| **#1** | MeSH descriptor: [Breast Feeding] explode all trees | 3094 |
| **#2** | (Breastfeeding or Breastfed or Chestfeeding or Breastfeeding or Breast Feedings or Chestfeedings or Breast Fed or Breastfeeding, Exclusive or Exclusive Breastfeeding or Exclusive Breast Feeding or Breast Feeding, Exclusive or Wet Nursing or Sharing, Milk or Milk Sharing):ti,ab,kw | 14398 |
| **#3** | #1 OR #2 | 14408 |
| **#4** | MeSH descriptor: [Artificial Intelligence] explode all trees | 4064 |
| **#5** | (Artificial Intelligence or Vision System, Computer or Systems, Computer Vision or Computer Vision System or Computer Vision Systems or System, Computer Vision or Vision Systems, Computer or AI (Artificial Intelligence) or Intelligence, Artificial or Computational Intelligence or Intelligence, Machine or Machine Intelligence or Computer Reasoning or Intelligence, Computational or Reasoning, Computer or Knowledge Acquisition (Computer) or Acquisition, Knowledge (Computer) or Knowledge Representation (Computer) or Representation, Knowledge (Computer) or Knowledge Representations (Computer)):ti,ab,kw | 8496 |
| **#6** | #4 OR #5 | 11360 |
| **#7** | MeSH descriptor: [Generative Artificial Intelligence] explode all trees | 78 |
| **#8** | (Generative Artificial Intelligence or Artificial Intelligence, Generative or Chatbot or Chatbots or ChatGPTs or Chat GPT or ChatGPT or Chat-GPT):ti,ab,kw | 1511 |
| **#9** | #7 OR #8 | 1511 |
| **#10** | MeSH descriptor: [Large Language Models] explode all trees | 28 |
| **#11** | (Large Language Model or Models, Large Language or Language Model, Large or Language Models, Large or Large Language Model or Model, Large Language):ti,ab,kw | 1184 |
| **#12** | #10 OR #11 | 1184 |
| **#13** | MeSH descriptor: [Machine Learning] explode all trees | 1344 |
| **#14** | (Machine Learning or Transfer Learning or Learning, Transfer or Learning, Machine):ti,ab,kw | 6435 |
| **#15** | #13 OR #14 | 6849 |
| **#16** | MeSH descriptor: [Machine Learning Algorithms] explode all trees | 1 |
| **#17** | (Machine Learning Algorithms or Algorithm, Machine Learning or Machine Learning Algorithm or Learning Algorithm, Machine):ti,ab,kw | 1569 |
| **#18** | #16 OR #17 | 1570 |
| **#19** | MeSH descriptor: [Deep Learning] explode all trees | 440 |
| **#20** | (Deep Learning or Hierarchical Learning or Learning, Deep or Learning, Hierarchical):ti,ab,kw | 2483 |
| **#21** | #19 OR #20 | 2510 |
| **#22** | MeSH descriptor: [Telemedicine] explode all trees | 6149 |
| **#23** | (Telemedicine or Medicine, Virtual or Tele-Referrals or Tele Referral or Virtual Medicine or Tele-Referral or Tele-Intensive Care or Tele ICU or Tele Intensive Care or Tele-ICU or Telecare or Tele-Care or Tele Care or Health, Mobile or mHealth or Mobile Health or Telehealth or eHealth):ti,ab,kw | 35174 |
| **#24** | #22 OR #23 | 35924 |
| **#25** | MeSH descriptor: [Mobile Applications] explode all trees | 2949 |
| **#26** | (Mobile Applications or Mobile Application or Apps, Mobile or Mobile Apps or App, Mobile or Application, Mobile or Applications, Mobile or Mobile App or Smartphone Apps or Apps, Smartphone or App, Smartphone or Smartphone App or Portable Electronic Application or Portable Electronic Apps or Electronic App, Portable or App, Portable Electronic or Portable Electronic Applications or Application, Portable Electronic or Electronic Application, Portable or Portable Electronic App or Software Application, Portable or Application, Portable Software or Portable Software Applications or Software App, Portable or Portable Software Apps or App, Portable Software or Portable Software App or Portable Software Application):ti,ab,kw | 17290 |
| **#27** | #25 OR #26 | 17290 |
| **#28** | MeSH descriptor: [Text Messaging] explode all trees | 1882 |
| **#29** | (Text Messaging or Messaging, Text or Texting or Short Message Service or Text Messages or Messages, Text or Message, Text or Text Message):ti,ab,kw | 18899 |
| **#30** | #28 OR #29 | 18899 |
| **#31** | MeSH descriptor: [Wearable Electronic Devices] explode all trees | 1250 |
| **#32** | (Wearable Electronic Devices or Device, Wearable Electronic or Electronic Device, Wearable or Wearable Electronic Device or Wearable Devices or Device, Wearable or Wearable Device or Wearable Technology or Technology, Wearable or Wearable Technologies or Electronic Skin or Skin, Electronic or Wearable Computer or Computer, Wearable or Wearable Computers):ti,ab,kw (Word variations have been searched) | 4270 |
| **#33** | #31 OR #32 | 5027 |
| **#34** | MeSH descriptor: [Virtual Reality] explode all trees | 1799 |
| **#35** | (Virtual Reality OR Virtual Reality, Instructional OR Virtual Realities, Instructional OR Instructional Virtual Reality OR Reality, Instructional Virtual OR Educational Virtual Reality OR Reality, Educational Virtual OR Virtual Reality, Educational OR Virtual Realities, Educational OR Educational Virtual Realities OR Instructional Virtual Realities OR Realities, Instructional Virtual OR Reality, Virtual):ti,ab,kw | 9991 |
| **#36** | #34 OR #35 | 10011 |
| **#37** | MeSH descriptor: [Internet-Based Intervention] explode all trees | 1100 |
| **#38** | (Internet-Based Intervention OR Intervention, Online OR Web-based Interventions OR Internet Interventions OR Interventions, Internet OR Intervention, Internet OR Interventions, Web-based OR Intervention, Web-based OR Online Interventions OR Internet Intervention OR Web-based Intervention OR Internet Based Intervention OR Internet-Based Interventions OR Interventions, Internet-Based OR Online Intervention OR Interventions, Online OR Intervention, Internet-Based OR Web based Intervention):ti,ab,kw | 36568 |
| **#39** | #37 OR #38 | 36568 |
| **#40** | #6 OR #9 OR #12 OR #15 OR #18 OR #21 OR #24 OR #27 OR #30 OR #33 OR #36 OR #39 | 151901 |
| **#41** | MeSH descriptor: [Randomized Controlled Trial] explode all trees | 34 |
| **#42** | (Randomized Controlled Trial or Randomized Controlled Trials as Topic or Randomized Controlled Trials as Topic or Clinical Trials, Randomized or Trials, Randomized Clinical or Controlled Clinical Trials,Randomized or Random Allocation or Allocation, Random or Randomization):ti,ab,kw | 1478437 |
| **#43** | #41 OR #42 | 1478437 |
| **#44** | MeSH descriptor: [Non-Randomized Controlled Trials as Topic] explode all trees | 317 |
| **#45** | (Non-Randomized Controlled Trials as Topic OR Non Randomized Controlled Trials as Topic OR Nonrandomized Controlled Trials as Topic OR Clinical Trials, Nonrandomized OR Clinical Trial, Nonrandomized OR Nonrandomized Clinical Trial OR Nonrandomized Clinical Trials OR Trial, Nonrandomized Clinical OR Trials, Nonrandomized Clinical OR Controlled Clinical Trials, Non-Randomized OR Controlled Clinical Trials, Non Randomized OR Quasi-Experimental Studies OR Quasi Experimental Studies OR Quasi-Experimental Study OR Studies, Quasi-Experimental OR Study, Quasi-Experimental OR Clinical Trials, Non-Randomized OR Clinical Trial, Non-Randomized OR Clinical Trials, Non Randomized OR Non-Randomized Clinical Trial OR Non-Randomized Clinical Trials OR Trial, Non-Randomized Clinical OR Trials, Non-Randomized Clinical OR Controlled Clinical Trials, Nonrandomized):ti,ab,kw | 174170 |
| **#46** | #44 OR #45 | 174170 |
| **#47** | #43 OR #46 | 1483963 |
| **#45** | #3 AND #40 AND #47 | 992 |

**Database: Embase**

|  | **Searches** | **Results** |
| --- | --- | --- |
| #1 | 'artificial intelligence'/exp | 199113 |
| #2 | 'machine intelligence':ab,ti OR 'artificial intelligence':ab,ti | 108559 |
| #3 | #1 OR #2 | 209673 |
| #4 | 'generative artificial intelligence'/exp | 21501 |
| #5 | 'creative ai':ab,ti OR 'creative artificial intelligence':ab,ti OR 'gen-ai':ab,ti OR 'genai':ab,ti OR 'generative a.i.':ab,ti OR 'generative ai':ab,ti OR 'generative artificial intelligence':ab,ti | 7998 |
| #6 | #4 OR #5 | 22553 |
| #7 | 'large language model'/exp | 20788 |
| #8 | 'large language models':ab,ti OR 'large language model':ab,ti | 13811 |
| #9 | #7 OR #8 | 22867 |
| #10 | 'machine learning'/exp | 738522 |
| #11 | 'learning machine':ab,ti OR 'learning machines':ab,ti OR 'machine learning':ab,ti | 200812 |
| #12 | #10 OR #11 | 756759 |
| #13 | 'deep learning'/exp | 114048 |
| #14 | 'deep machine learning':ab,ti OR 'deep ml':ab,ti OR 'hierarchical learning':ab,ti OR 'deep learning':ab,ti | 113039 |
| #15 | #13 OR #14 | 136703 |
| #16 | 'telemedicine'/exp | 97068 |
| #17 | 'tele medicine':ab,ti OR 'virtual medicine':ab,ti OR 'telemedicine':ab,ti | 35482 |
| #18 | #16 OR #17 | 101977 |
| #19 | 'mobile application'/exp | 40715 |
| #20 | 'mobile app':ab,ti OR 'mobile applications':ab,ti OR 'mobile apps':ab,ti OR 'portable software app':ab,ti OR 'portable software application':ab,ti OR 'portable software applications':ab,ti OR 'portable software apps':ab,ti OR 'tablet application':ab,ti OR 'mobile application':ab,ti | 19592 |
| #21 | #19 OR #20 | 43606 |
| #22 | 'text messaging'/exp | 12864 |
| #23 | 'texting':ab,ti OR 'text messaging':ab,ti | 6863 |
| #24 | #22 OR #23 | 14132 |
| #25 | 'wearable electronic device'/exp | 30154 |
| #26 | 'electronic wearable':ab,ti OR 'electronic wearables':ab,ti OR 'wearable electronic':ab,ti OR 'wearable electronic devices':ab,ti OR 'wearable electronical device':ab,ti OR 'wearable electronics':ab,ti OR 'wearable electronic device':ab,ti | 3572 |
| #27 | #25 OR #26 | 32641 |
| #28 | 'Virtual reality'/exp | 41447 |
| #29 | 'Virtual reality':ab,ti | 33159 |
| #30 | #28 OR #29 |  |
| #31 | 'web-based intervention'/exp | 6231 |
| #32 | 'internet-based intervention'/exp OR 'internet-based intervention' OR 'internet-intervention'/exp OR 'internet-intervention' OR 'online-based intervention'/exp OR 'online-based intervention' OR 'online-intervention'/exp OR 'online-intervention' OR 'web intervention'/exp OR 'web intervention' OR 'web-based intervention'/exp OR 'web-based intervention' | 9248 |
| #33 | #31 OR #32 | 9248 |
| #34 | #5 OR #6 OR #9 OR #12 OR #15 OR #18 OR #21 OR #26 OR #27 OR #30 OR #33 | 1080429 |
| #35 | 'breast feeding'/exp | 83478 |
| #36 | 'feeding, breast':ab,ti OR 'breast feeding':ab,ti | 18319 |
| #37 | #35 OR #36 | 87187 |
| #38 | 'randomized controlled trial'/exp | 1177282 |
| #39 | 'controlled trial, randomized':ab,ti OR 'randomised controlled study':ab,ti OR 'randomised controlled trial':ab,ti OR 'randomized controlled study':ab,ti OR 'trial, randomized controlled':ab,ti OR 'randomized controlled trial':ab,ti | 302224 |
| #40 | #38 OR #39 | 1224980 |
| #41 | 'controlled clinical trial'/exp | 1368675 |
| #42 | 'clinical trial, controlled':ab,ti OR 'controlled clinical comparison':ab,ti OR 'controlled clinical drug trial':ab,ti OR 'controlled clinical experiment':ab,ti OR 'controlled clinical study':ab,ti OR 'controlled clinical test':ab,ti OR 'controlled clinical trial':ab,ti | 44077 |
| #43 | #41 OR #42 | 1376553 |
| #44 | #40 OR #43 | 1422707 |
| #45 | #34 AND #37 AND #44 | 158 |

**Database: PubMed**

|  | **Searches** | **Results** |
| --- | --- | --- |
| #1 | **("Breast Feeding"[Mesh]) OR ((((((((((((Breast Feedings[Title/Abstract]) OR (Breast Fed[Title/Abstract])) OR (Breastfed[Title/Abstract])) OR (Chestfeeding[Title/Abstract])) OR (Breastfeeding[Title/Abstract])) OR (Breast Feeding, Exclusive[Title/Abstract])) OR (Exclusive Breast Feeding[Title/Abstract])) OR (Breastfeeding, Exclusive[Title/Abstract])) OR (Exclusive Breastfeeding[Title/Abstract])) OR (Wet Nursing[Title/Abstract])) OR (Milk Sharing[Title/Abstract])) OR (Sharing, Milk[Title/Abstract]))** | 74861 |
| #2 | ("Artificial Intelligence"[Mesh]) OR (((((((((Artificial Intelligence[Title/Abstract]) OR (Intelligence, Artificial[Title/Abstract])) OR (Computer Reasoning[Title/Abstract])) OR (Reasoning, Computer[Title/Abstract])) OR (AI (Artificial Intelligence[Title/Abstract]))) OR (Machine Intelligence[Title/Abstract])) OR (Intelligence, Machine[Title/Abstract])) OR (Computational Intelligence[Title/Abstract])) OR (Intelligence, Computational[Title/Abstract])) | 335741 |
| #3 | ("Generative Artificial Intelligence"[Mesh]) OR (((((((Artificial Intelligence, Generative[Title/Abstract]) OR (Chatbot[Title/Abstract])) OR (Chatbots[Title/Abstract])) OR (Chat-GPT[Title/Abstract])) OR (Chat GPT[Title/Abstract])) OR (ChatGPT[Title/Abstract])) OR (ChatGPTs[Title/Abstract])) | 12742 |
| #4 | ("Large Language Models"[Mesh]) OR (((((Language Model, Large[Title/Abstract]) OR (Language Models, Large[Title/Abstract])) OR (Large Language Model[Title/Abstract])) OR (Model, Large Language[Title/Abstract])) OR (Models, Large Language[Title/Abstract])) | 6036 |
| #5 | ("Machine Learning"[Mesh]) OR (((Learning, Machine[Title/Abstract]) OR (Transfer Learning[Title/Abstract])) OR (Learning, Transfer[Title/Abstract])) | 128027 |
| #6 | ("Deep Learning"[Mesh]) OR (Learning, Deep[Title/Abstract]) OR (Hierarchical Learning[Title/Abstract]) | 41756 |
| #7 | ("Neural Networks, Computer"[Mesh]) OR (((((((((((((((((((((((((((Computer Neural Network[Title/Abstract]) OR (Computer Neural Networks[Title/Abstract])) OR (Network, Computer Neural[Title/Abstract])) OR (Networks, Computer Neural[Title/Abstract])) OR (Neural Network, Computer[Title/Abstract])) OR (Models, Neural Network[Title/Abstract])) OR (Model, Neural Network[Title/Abstract])) OR (Network Model, Neural[Title/Abstract])) OR (Network Models, Neural[Title/Abstract])) OR (Neural Network Model[Title/Abstract])) OR (Neural Network Models[Title/Abstract])) OR (Connectionist Models[Title/Abstract])) OR (Connectionist Model[Title/Abstract])) OR (Model, Connectionist[Title/Abstract])) OR (Models, Connectionist[Title/Abstract])) OR (Perceptrons[Title/Abstract])) OR (Perceptron[Title/Abstract])) OR (Computational Neural Networks[Title/Abstract])) OR (Computational Neural Network[Title/Abstract])) OR (Network, Computational Neural[Title/Abstract])) OR (Networks, Computational Neural[Title/Abstract])) OR (Neural Network, Computational[Title/Abstract])) OR (Neural Networks, Computational[Title/Abstract])) OR (Neural Networks (Computer[Title/Abstract]))) OR (Network, Neural (Computer[Title/Abstract]))) OR (Networks, Neural (Computer[Title/Abstract]))) OR (Neural Network (Computer[Title/Abstract]))) | 117891 |
| #8 | **("Machine Learning Algorithms"[Mesh]) OR (((Algorithm, Machine Learning[Title/Abstract]) OR (Learning Algorithm, Machine[Title/Abstract])) OR (Machine Learning Algorithm[Title/Abstract]))** | 7275 |
| #9 | ("Telemedicine"[Mesh]) OR (((((((((((((((((Virtual Medicine[Title/Abstract]) OR (Medicine, Virtual[Title/Abstract])) OR (Tele-Referral[Title/Abstract])) OR (Tele Referral[Title/Abstract])) OR (Tele-Referrals[Title/Abstract])) OR (Mobile Health[Title/Abstract])) OR (Health, Mobile[Title/Abstract])) OR (mHealth[Title/Abstract])) OR (Telehealth[Title/Abstract])) OR (eHealth[Title/Abstract])) OR (Tele-Intensive Care[Title/Abstract])) OR (Tele Intensive Care[Title/Abstract])) OR (Tele-ICU[Title/Abstract])) OR (Tele ICU[Title/Abstract])) OR (Telecare[Title/Abstract])) OR (Tele-Care[Title/Abstract])) OR (Tele Care[Title/Abstract])) | 86973 |
| #10 | ("Mobile Applications"[Mesh]) OR (((((((((((((((((((((((((((Application, Mobile[Title/Abstract]) OR (Applications, Mobile[Title/Abstract])) OR (Mobile Application[Title/Abstract])) OR (Mobile Apps[Title/Abstract])) OR (App, Mobile[Title/Abstract])) OR (Apps, Mobile[Title/Abstract])) OR (Mobile App[Title/Abstract])) OR (Portable Electronic Apps[Title/Abstract])) OR (App, Portable Electronic[Title/Abstract])) OR (Electronic App, Portable[Title/Abstract])) OR (Portable Electronic App[Title/Abstract])) OR (Portable Electronic Applications[Title/Abstract])) OR (Application, Portable Electronic[Title/Abstract])) OR (Electronic Application, Portable[Title/Abstract])) OR (Portable Electronic Application[Title/Abstract])) OR (Portable Software Apps[Title/Abstract])) OR (App, Portable Software[Title/Abstract])) OR (Portable Software App[Title/Abstract])) OR (Software App, Portable[Title/Abstract])) OR (Portable Software Applications[Title/Abstract])) OR (Application, Portable Software[Title/Abstract])) OR (Portable Software Application[Title/Abstract])) OR (Software Application, Portable[Title/Abstract])) OR (Smartphone Apps[Title/Abstract])) OR (App, Smartphone[Title/Abstract])) OR (Apps, Smartphone[Title/Abstract])) OR (Smartphone App[Title/Abstract])) | 27147 |
| #11 | (((((((((((((((((((((((((("Virtual Reality"[Mesh]) OR (Virtual Reality[Title/Abstract])) OR (Reality, Virtual[Title/Abstract])) OR (Virtual Reality, Educational[Title/Abstract])) OR (Educational Virtual Realities[Title/Abstract])) OR (Educational Virtual Reality[Title/Abstract])) OR (Reality, Educational Virtual[Title/Abstract])) OR (Virtual Realities, Educational[Title/Abstract])) OR (Virtual Reality, Instructional[Title/Abstract])) OR (Instructional Virtual Realities[Title/Abstract])) OR (Instructional Virtual Reality[Title/Abstract])) OR (Realities, Instructional Virtual[Title/Abstract])) OR (Reality, Instructional Virtual[Title/Abstract])) OR (Virtual Realities, Instructional[Title/Abstract])) OR (digital technology[Title/Abstract])) OR (new technology[Title/Abstract])) OR (immersive multimedia[Title/Abstract])) OR (virtual environment[Title/Abstract])) OR (video game[Title/Abstract])) OR (virtual game[Title/Abstract])) OR (virtual simulation[Title/Abstract])) OR (virtual patient[Title/Abstract])) OR (virtual patients[Title/Abstract])) OR (patient simulation[Title/Abstract])) OR (patient simulations[Title/Abstract])) OR (virtual reality simulation devices[Title/Abstract]) OR (virtual reality simulation system[Title/Abstract]) OR (VR simulation system[Title/Abstract]) OR (VR simulator[Title/Abstract]) OR (virtual reality simulator[Title/Abstract])) | 58058 |
| #12 | **("Wearable Electronic Devices"[Mesh]) OR ((((((((((((((Device, Wearable Electronic[Title/Abstract]) OR (Electronic Device, Wearable[Title/Abstract])) OR (Wearable Electronic Device[Title/Abstract])) OR (Wearable Devices[Title/Abstract])) OR (Device, Wearable[Title/Abstract])) OR (Wearable Device[Title/Abstract])) OR (Wearable Technology[Title/Abstract])) OR (Technology, Wearable[Title/Abstract])) OR (Wearable Technologies[Title/Abstract])) OR (Electronic Skin[Title/Abstract])) OR (Skin, Electronic[Title/Abstract])) OR (Wearable Computer[Title/Abstract])) OR (Computer, Wearable[Title/Abstract])) OR (Wearable Computers[Title/Abstract]))** | 42152 |
| #13 | (("Text Messaging"[Mesh]) OR ((((((((Messaging, Text[Title/Abstract]) OR (Texting[Title/Abstract])) OR (Short Message Service[Title/Abstract])) OR (Text Messages[Title/Abstract])) OR (Messages, Text[Title/Abstract])) OR (Message, Text[Title/Abstract])) OR (Text Message[Title/Abstract])) OR (Text Messaging[Title/Abstract]))) | 11527 |
| #14 | **(("Internet-Based Intervention"[Mesh]) OR ((((((((((((((((((Internet-Based Intervention[Title/Abstract]) OR (Internet Based Intervention[Title/Abstract])) OR (Internet-Based Interventions[Title/Abstract])) OR (Intervention, Internet-Based[Title/Abstract])) OR (Interventions, Internet-Based[Title/Abstract])) OR (Internet Intervention[Title/Abstract])) OR (Internet Interventions[Title/Abstract])) OR (Intervention, Internet[Title/Abstract])) OR (Interventions, Internet[Title/Abstract])) OR (Web-based Intervention[Title/Abstract])) OR (Interventions, Web-based[Title/Abstract])) OR (Intervention, Web-based[Title/Abstract])) OR (Web based Intervention[Title/Abstract])) OR (Web-based Interventions[Title/Abstract])) OR (Online Intervention[Title/Abstract])) OR (Intervention, Online[Title/Abstract])) OR (Interventions, Online[Title/Abstract])) OR (Online Interventions[Title/Abstract])))** | 7279 |
| #15 | #2 OR #3 OR #4 OR #5 OR #6 OR #7 OR #8 OR #9 OR #10 OR #11 OR #12 OR #13 OR #14 | 556563 |
| #16 | (("Randomized Controlled Trial" [Publication Type]) OR (("Randomized Controlled Trials as Topic"[Mesh]) OR ((((Randomized Controlled Trials as Topic[Title/Abstract]) OR (Clinical Trials, Randomized[Title/Abstract])) OR (Trials, Randomized Clinical[Title/Abstract])) OR (Controlled Clinical Trials, Randomized[Title/Abstract])))) OR (("Random Allocation"[Mesh]) OR (((Random Allocation[Title/Abstract]) OR (Allocation, Random[Title/Abstract])) OR (Randomization[Title/Abstract]))) | 977344 |
| #17 | **("Non-Randomized Controlled Trials as Topic"[Mesh]) OR (((((((((((((((((((((((Non Randomized Controlled Trials as Topic[Title/Abstract]) OR (Nonrandomized Controlled Trials as Topic[Title/Abstract])) OR (Clinical Trials, Nonrandomized[Title/Abstract])) OR (Clinical Trial, Nonrandomized[Title/Abstract])) OR (Nonrandomized Clinical Trial[Title/Abstract])) OR (Nonrandomized Clinical Trials[Title/Abstract])) OR (Trial, Nonrandomized Clinical[Title/Abstract])) OR (Trials, Nonrandomized Clinical[Title/Abstract])) OR (Controlled Clinical Trials, Non-Randomized[Title/Abstract])) OR (Controlled Clinical Trials, Non Randomized[Title/Abstract])) OR (Quasi-Experimental Studies[Title/Abstract])) OR (Quasi Experimental Studies[Title/Abstract])) OR (Quasi-Experimental Study[Title/Abstract])) OR (Studies, Quasi-Experimental[Title/Abstract])) OR (Study, Quasi-Experimental[Title/Abstract])) OR (Clinical Trials, Non-Randomized[Title/Abstract])) OR (Clinical Trial, Non-Randomized[Title/Abstract])) OR (Clinical Trials, Non Randomized[Title/Abstract])) OR (Non-Randomized Clinical Trial[Title/Abstract])) OR (Non-Randomized Clinical Trials[Title/Abstract])) OR (Trial, Non-Randomized Clinical[Title/Abstract])) OR (Trials, Non-Randomized Clinical[Title/Abstract])) OR (Controlled Clinical Trials, Nonrandomized[Title/Abstract]))** | 15512 |
| #18 | #16 OR #17 | 998838 |
| #18 | #1 AND #15 AND #18 | 551 |

**Database: Web of Science**

|  | **Searches** | **Results** |
| --- | --- | --- |
| #1 | (TI=(Breastfeeding OR Breast Feedings OR Breast Fed OR Breastfed OR Chestfeedings OR Breastfeeding OR Breast Feeding, Exclusive OR Exclusive Breast Feeding OR Breastfeeding, Exclusive OR Exclusive Breastfeeding OR Wet Nursing OR Milk Sharing OR Sharing, Milk)) OR (AB=(Breastfeeding OR Breast Feedings OR Breast Fed OR Breastfed OR Chestfeedings OR Breastfeeding OR Breast Feeding, Exclusive OR Exclusive Breast Feeding OR Breastfeeding, Exclusive OR Exclusive Breastfeeding OR Wet Nursing OR Milk Sharing OR Sharing, Milk)) | 120000 |
| #2 | (TI=(Artificial Intelligence OR Intelligence, Artificial OR Computer Reasoning OR Reasoning, Computer OR AI OR Machine Intelligence OR Intelligence, Machine OR Computational Intelligence OR Intelligence, Computational)) OR (AB=(Artificial Intelligence OR Intelligence, Artificial OR Computer Reasoning OR Reasoning, Computer OR AI OR Machine Intelligence OR Intelligence, Machine OR Computational Intelligence OR Intelligence, Computational)) | 730852 |
| #3 | (TI=(Generative Artificial Intelligence or Artificial Intelligence, Generative or Chatbot or Chatbots or ChatGPTs or Chat GPT or ChatGPT or Chat-GPT)) OR (AB=(Generative Artificial Intelligence or Artificial Intelligence, Generative or Chatbot or Chatbots or ChatGPTs or Chat GPT or ChatGPT or Chat-GPT)) | 63303 |
| #4 | (TI=(Large Language Model or Models, Large Language or Language Model, Large or Language Models, Large or Large Language Model or Model, Large Language)) OR (AB=(Large Language Model or Models, Large Language or Language Model, Large or Language Models, Large or Large Language Model or Model, Large Language)) | 154617 |
| #5 | (TI=(Machine Learning or Transfer Learning or Learning, Transfer or Learning, Machine)) OR (AB=(Machine Learning or Transfer Learning or Learning, Transfer or Learning, Machine)) | 846630 |
| #6 | (TI=(Deep Learning or Hierarchical Learning or Learning, Deep or Learning, Hierarchical)) OR (AB=(Deep Learning or Hierarchical Learning or Learning, Deep or Learning, Hierarchical)) | 616191 |
| #7 | (TI=(Wearable Electronic Devices or Device, Wearable Electronic or Electronic Device, Wearable or Wearable Electronic Device or Wearable Devices or Device, Wearable or Wearable Device or Wearable Technology or Technology, Wearable or Wearable Technologies or Electronic Skin or Skin, Electronic or Wearable Computer or Computer, Wearable or Wearable Computers)) OR (AB=(Wearable Electronic Devices or Device, Wearable Electronic or Electronic Device, Wearable or Wearable Electronic Device or Wearable Devices or Device, Wearable or Wearable Device or Wearable Technology or Technology, Wearable or Wearable Technologies or Electronic Skin or Skin, Electronic or Wearable Computer or Computer, Wearable or Wearable Computers)) | 87986 |
| #8 | (TI=(Telemedicine or Medicine, Virtual or Tele-Referrals or Tele Referral or Virtual Medicine or Tele-Referral or Tele-Intensive Care or Tele ICU or Tele Intensive Care or Tele-ICU or Telecare or Tele-Care or Tele Care or Health, Mobile or mHealth or Mobile Health or Telehealth or eHealth)) OR (AB=(Telemedicine or Medicine, Virtual or Tele-Referrals or Tele Referral or Virtual Medicine or Tele-Referral or Tele-Intensive Care or Tele ICU or Tele Intensive Care or Tele-ICU or Telecare or Tele-Care or Tele Care or Health, Mobile or mHealth or Mobile Health or Telehealth or eHealth)) | 154172 |
| #9 | (TI=(Mobile Applications or Mobile Application or Apps, Mobile or Mobile Apps or App, Mobile or Application, Mobile or Applications, Mobile or Mobile App or Smartphone Apps or Apps, Smartphone or App, Smartphone or Smartphone App or Portable Electronic Application or Portable Electronic Apps or Electronic App, Portable or App, Portable Electronic or Portable Electronic Applications or Application, Portable Electronic or Electronic Application, Portable or Portable Electronic App or Software Application, Portable or Application, Portable Software or Portable Software Applications or Software App, Portable or Portable Software Apps or App, Portable Software or Portable Software App or Portable Software Application)) OR (AB=(Mobile Applications or Mobile Application or Apps, Mobile or Mobile Apps or App, Mobile or Application, Mobile or Applications, Mobile or Mobile App or Smartphone Apps or Apps, Smartphone or App, Smartphone or Smartphone App or Portable Electronic Application or Portable Electronic Apps or Electronic App, Portable or App, Portable Electronic or Portable Electronic Applications or Application, Portable Electronic or Electronic Application, Portable or Portable Electronic App or Software Application, Portable or Application, Portable Software or Portable Software Applications or Software App, Portable or Portable Software Apps or App, Portable Software or Portable Software App or Portable Software Application)) | 201218 |
| #10 | (TI=(Text Messaging OR Messaging, Text OR Texting OR Short Message Service OR Text Messages OR Messages, Text OR Message, Text OR Text Message)) OR (AB=(Text Messaging OR Messaging, Text OR Texting OR Short Message Service OR Text Messages OR Messages, Text OR Message, Text OR Text Message)) | 44357 |
| #11 | (TI=(Virtual Reality OR Reality, Virtual OR Virtual Reality, Educational OR Educational Virtual Realities OR Educational Virtual Reality OR Reality, Educational Virtual OR Virtual Realities, Educational OR Virtual Reality, Instructional OR Instructional Virtual Realities OR Instructional Virtual Reality OR Realities, Instructional Virtual OR Reality, Instructional Virtual OR Virtual Realities, Instructional OR digital technology OR new technology OR immersive multimedia OR virtual environment OR video game OR virtual game OR virtual simulation OR virtual patient OR virtual patients OR patient simulation OR patient simulations OR virtual reality simulation devices OR virtual reality simulation system OR VR simulation system OR VR simulator OR virtual reality simulator)) OR (AB=(Virtual Reality OR Reality, Virtual OR Virtual Reality, Educational OR Educational Virtual Realities OR Educational Virtual Reality OR Reality, Educational Virtual OR Virtual Realities, Educational OR Virtual Reality, Instructional OR Instructional Virtual Realities OR Instructional Virtual Reality OR Realities, Instructional Virtual OR Reality, Instructional Virtual OR Virtual Realities, Instructional OR digital technology OR new technology OR immersive multimedia OR virtual environment OR video game OR virtual game OR virtual simulation OR virtual patient OR virtual patients OR patient simulation OR patient simulations OR virtual reality simulation devices OR virtual reality simulation system OR VR simulation system OR VR simulator OR virtual reality simulator)) | 1540887 |
| #12 | **(TI=(Internet-Based Intervention OR Internet Based Intervention OR Internet-Based Interventions OR Intervention, Internet-Based OR Interventions, Internet-Based OR Internet Intervention OR Internet Interventions OR Intervention, Internet OR Interventions, Internet OR Web-based Intervention OR Interventions, Web-based OR Intervention, Web-based OR Web based Intervention OR Web-based Interventions OR Online Intervention OR Intervention, Online OR Interventions, Online OR Online Interventions)) OR (AB=(Internet-Based Intervention OR Internet Based Intervention OR Internet-Based Interventions OR Intervention, Internet-Based OR Interventions, Internet-Based OR Internet Intervention OR Internet Interventions OR Intervention, Internet OR Interventions, Internet OR Web-based Intervention OR Interventions, Web-based OR Intervention, Web-based OR Web based Intervention OR Web-based Interventions OR Online Intervention OR Intervention, Online OR Interventions, Online OR Online Interventions))** | 58176 |
| #13 | #2 OR #3 OR #4 OR #5 OR #6 OR #7 OR #8 OR #9 OR #10 OR #11 OR #12 | 4004497 |
| #14 | (TI=(Randomized Controlled Trial or Randomized Controlled Trials as Topic or Randomized Controlled Trials as Topic or Clinical Trials, Randomized or Trials, Randomized Clinical or Controlled Clinical Trials,Randomized or Random Allocation or Allocation, Random or Randomization)) OR (AB=(Randomized Controlled Trial or Randomized Controlled Trials as Topic or Randomized Controlled Trials as Topic or Clinical Trials, Randomized or Trials, Randomized Clinical or Controlled Clinical Trials,Randomized or Random Allocation or Allocation, Random or Randomization)) | 871602 |
| #15 | (TI=(Non-Randomized Controlled Trials as Topic OR Non Randomized Controlled Trials as Topic OR Nonrandomized Controlled Trials as Topic OR Clinical Trials, Nonrandomized OR Clinical Trial, Nonrandomized OR Nonrandomized Clinical Trial OR Nonrandomized Clinical Trials OR Trial, Nonrandomized Clinical OR Trials, Nonrandomized Clinical OR Controlled Clinical Trials, Non-Randomized OR Controlled Clinical Trials, Non Randomized OR Quasi-Experimental Studies OR Quasi Experimental Studies OR Quasi-Experimental Study OR Studies, Quasi-Experimental OR Study, Quasi-Experimental OR Clinical Trials, Non-Randomized OR Clinical Trial, Non-Randomized OR Clinical Trials, Non Randomized OR Non-Randomized Clinical Trial OR Non-Randomized Clinical Trials OR Trial, Non-Randomized Clinical OR Trials, Non-Randomized Clinical OR Controlled Clinical Trials, Nonrandomized)) OR (AB=(Non-Randomized Controlled Trials as Topic OR Non Randomized Controlled Trials as Topic OR Nonrandomized Controlled Trials as Topic OR Clinical Trials, Nonrandomized OR Clinical Trial, Nonrandomized OR Nonrandomized Clinical Trial OR Nonrandomized Clinical Trials OR Trial, Nonrandomized Clinical OR Trials, Nonrandomized Clinical OR Controlled Clinical Trials, Non-Randomized OR Controlled Clinical Trials, Non Randomized OR Quasi-Experimental Studies OR Quasi Experimental Studies OR Quasi-Experimental Study OR Studies, Quasi-Experimental OR Study, Quasi-Experimental OR Clinical Trials, Non-Randomized OR Clinical Trial, Non-Randomized OR Clinical Trials, Non Randomized OR Non-Randomized Clinical Trial OR Non-Randomized Clinical Trials OR Trial, Non-Randomized Clinical OR Trials, Non-Randomized Clinical OR Controlled Clinical Trials, Nonrandomized)) | 196834 |
| #16 | #14 OR #15 | 995167 |
| #17 | #1 AND #13 AND #16 | 5990 |

**Database: Scopus**

|  | **Searches** | **Results** |
| --- | --- | --- |
| #1 | ("Breast Feeding" OR Breastfeeding OR Breastfed OR Chestfeeding OR Breastfeeding OR "Breast Feedings" OR Chestfeedings OR "Breast Fed" OR "Breastfeeding, Exclusive" OR "Exclusive Breastfeeding" OR "Exclusive Breast Feeding" OR "Breast Feeding, Exclusive" OR "Wet Nursing" OR "Sharing, Milk" OR "Milk Sharing") | 103105 |
| #2 | ("Artificial Intelligence" OR "Vision System, Computer" OR "Systems, Computer Vision" OR "Computer Vision System" OR "Computer Vision Systems" OR "System, Computer Vision" OR "Vision Systems, Computer" OR "AI (Artificial Intelligence)" OR "Intelligence, Artificial" OR "Computational Intelligence" OR "Intelligence, Machine" OR "Machine Intelligence" OR "Computer Reasoning" OR "Intelligence, Computational" OR "Reasoning, Computer" OR "Knowledge Acquisition (Computer)" OR "Acquisition, Knowledge (Computer)" OR "Knowledge Representation (Computer)" OR "Representation, Knowledge (Computer)" OR "Knowledge Representations (Computer)") | 869512 |
| #3 | ("Generative Artificial Intelligence" OR "Artificial Intelligence, Generative" OR "Chatbot" OR "Chatbots" OR "ChatGPTs" OR "Chat GPT" OR "ChatGPT" OR "Chat-GPT") | 66375 |
| #4 | ( "Large Language Model" OR "Large Language Models" OR "Large Language" OR "Language Model, Large" OR "Language Models, Large" OR "Large Language Model" OR "Model, Large Language" ) | 87635 |
| #5 | ( "Machine Learning" OR "Transfer Learning" OR "Learning, Transfer" OR "Learning, Machine" ) | 1122883 |
| #6 | ( "Machine Learning Algorithms" OR "Algorithm, Machine Learning" OR "Machine Learning Algorithm" OR "Learning Algorithm, Machine" ) | 133482 |
| #7 | ( "Deep Learning" OR "Hierarchical Learning" OR "Learning, Deep" OR "Learning, Hierarchical" ) | 718040 |
| #8 | ( "Telemedicine" OR "Medicine, Virtual" OR "Tele-Referrals" OR "Tele Referral" OR "Virtual Medicine" OR "Tele-Referral" OR "Tele-Intensive Care" OR "Tele ICU" OR "Tele Intensive Care" OR "Tele-ICU" OR "Telecare" OR "Tele-Care" OR "Tele Care" OR "Health, Mobile" OR "mHealth" OR "Mobile Health" OR "Telehealth" OR "eHealth" ) | 160065 |
| #9 | ( "Mobile Applications" OR "Mobile Application" OR "Apps, Mobile" OR "Mobile Apps" OR "App, Mobile" OR "Application, Mobile" OR "Applications, Mobile" OR "Mobile App" OR "Smartphone Apps" OR "Apps, Smartphone" OR "App, Smartphone" OR "Smartphone App" OR "Portable Electronic Application" OR "Portable Electronic Apps" OR "Electronic App, Portable" OR "App, Portable Electronic" OR "Portable Electronic Applications" OR "Application, Portable Electronic" OR "Electronic Application, Portable" OR "Portable Electronic App" OR "Software Application, Portable" OR "Application, Portable Software" OR "Portable Software Applications" OR "Software App, Portable" OR "Portable Software Apps" OR "App, Portable Software" OR "Portable Software App" OR "Portable Software Application" ) | 102167 |
| #10 | ( "Text Messaging" OR "Messaging, Text" OR "Texting" OR "Short Message Service" OR "Text Messages" OR "Messages, Text" OR "Message, Text" OR "Text Message" ) | 25185 |
| #11 | ( "Wearable Electronic Devices" OR "Device, Wearable Electronic" OR "Electronic Device, Wearable" OR "Wearable Electronic Device" OR "Wearable Devices" OR "Device, Wearable" OR "Wearable Device" OR "Wearable Technology" OR "Technology, Wearable" OR "Wearable Technologies" OR "Electronic Skin" OR "Skin, Electronic" OR "Wearable Computer" OR "Computer, Wearable" OR "Wearable Computers" ) | 91200 |
| #12 | ( "Virtual Reality" OR "Reality, Virtual" OR "Virtual Reality, Educational" OR "Educational Virtual Realities" OR "Educational Virtual Reality" OR "Reality, Educational Virtual" OR "Virtual Realities, Educational" OR "Virtual Reality, Instructional" OR "Instructional Virtual Realities" OR "Instructional Virtual Reality" OR "Realities, Instructional Virtual" OR "Reality, Instructional Virtual" OR "Virtual Realities, Instructional" OR digital technology OR new technology OR "immersive multimedia" OR "virtual environment" OR "video game" OR "virtual game" OR "virtual simulation" OR "virtual patient" OR "virtual patients" OR "patient simulation" OR "patient simulations" OR "virtual reality simulation devices" OR "virtual reality simulation system" OR "VR simulation system" OR "VR simulator" OR "virtual reality simulator" ) | 562224 |
| #13 | #1 OR #2 OR #3 OR #4 OR #5 OR #6 OR #7 OR #8 OR #9 OR #10 OR #11 OR #12 | 3099573 |
| #14 | ( "Randomized Controlled Trial" OR "Randomized Controlled Trials as Topic" OR "Randomized Controlled Trials as Topic" OR "Clinical Trials, Randomized" OR "Trials, Randomized Clinical" OR "Controlled Clinical Trials,Randomized" OR "Random Allocation" OR "Allocation, Random" OR "Randomization" ) | 1343494 |
| #15 | ( "Non-Randomized Controlled Trials as Topic" OR "Non Randomized Controlled Trials as Topic" OR "Nonrandomized Controlled Trials as Topic" OR "Clinical Trials, Nonrandomized" OR "Clinical Trial, Nonrandomized" OR "Nonrandomized Clinical Trial" OR "Nonrandomized Clinical Trials" OR "Trial, Nonrandomized Clinical" OR "Trials, Nonrandomized Clinical" OR "Controlled Clinical Trials, Non-Randomized" OR "Controlled Clinical Trials, Non Randomized" OR "Quasi-Experimental Studies" OR "Quasi Experimental Studies" OR "Quasi-Experimental Study" OR "Studies, Quasi-Experimental" OR "Study, Quasi-Experimental" OR "Clinical Trials, Non-Randomized" OR "Clinical Trial, Non-Randomized" OR "Clinical Trials, Non Randomized" OR "Non-Randomized Clinical Trial" OR "Non-Randomized Clinical Trials" OR "Trial, Non-Randomized Clinical" OR "Trials, Non-Randomized Clinical" OR "Controlled Clinical Trials, Nonrandomized" ) | 31508 |
| #16 | #14 OR #15 | 1369348 |
| #17 | #1 AND #13 AND #16 | 272 |

**Database: IEEE Xplore Digital Library**

|  | **Searches** | **Results** |
| --- | --- | --- |
| #1 | ("All Metadata":Breast Feeding OR "All Metadata":Breastfeeding OR "All Metadata":Breast Feedings OR "All Metadata":Breast Fed) | 471 |
| #2 | ("All Metadata":Artificial Intelligence) | 359361 |
| #3 | ("All Metadata":Generative Artificial Intelligence or Artificial Intelligence, Generative) | 1337 |
| #4 | ("All Metadata":large language models or llm or nlp or natural language processing) | 672 |
| #5 | ("All Metadata":Machine Learning) | 493162 |
| #6 | ("All Metadata":Deep Learning) | 390081 |
| #7 | ("All Metadata":Machine Learning Algorithms) | 261700 |
| #8 | ("All Metadata":Wearable Electronic Devices) | 8014 |
| #9 | ("All Metadata":Telemedicine) | 10018 |
| #10 | ("All Metadata":Mobile Applications) | 205600 |
| #11 | ("All Metadata":Text Messaging) | 11745 |
| #12 | ("All Metadata":Virtual Reality) | 60719 |
| #13 | #2 OR #3 OR #4 OR #5 OR #6 OR #7 OR #8 OR #9 OR #10 OR #11 OR #12 | 1152972 |
| #14 | ("All Metadata":Randomized Controlled Trial or Randomized Controlled Trials as Topic) | 35 |
| #15 | ("All Metadata":Non-Randomized Controlled Trials as Topic or Nonrandomized Trials) | 0 |
| #16 | #15 OR #16 | 35 |
| #17 | #1 AND #13 AND #16 | 11 |
