# Supplementary material for: Effects of Digital Health Interventions on Breastfeeding Rates, Self-Efficacy, and Knowledge: Systematic Review and Meta-Analysis
Source: J Med Internet Res. 2026 Aug 12;28:e92184. doi: 10.2196/92184 (PMC13519512; doi:10.2196/92184)
Supplement: Supplementary file 4 [file jmir_v28i1e92184_app4.docx]

Table 1.

Inclusion of study characteristics of 11,704 lactating women from multicenter randomized controlled trials and quasi-experimental studies (2015–2026).

| Study, year, country, and study design | Study  Design | Partici-  pants | Contents of the intervention group | Follow-up | Outcome and outcome measurement tools |
| --- | --- | --- | --- | --- | --- |
| Acar et al [78], 2024, Turkey | RCT^a^ | 80  I^b^: 40  C^c^:40 | - Type: mHealth^d^（app） - Supported by mobile terminal data collection modules. Monitor breastfeeding status; monthly record breastfeeding frequency/duration, milking condition, infant height and weight, and breastfeeding-related problems. - Period: postnatal. - Frequency：no mention. - Duration: 2 months. | 4 weeks  8 weeks | EBF^e^ |
| Ahmed et at[79], 2016, USA | RCT | 106  I: 49  C: 57 | - Type: eHealth^f^ (telemedicine) - Once mothers submit data indicating breastfeeding issues, the system automatically sends feedback via notifications and provides tailored interventions. - Period: postnatal. - Frequency: 3 times per day. - Duration: 10 days. | 1 month  2 months  3 months | EBF |
| Araban et al[81], 2025, Turkey | RCT | 110  I:56  C:54 | - Type: mHealth (SMS^g^) - Provide positive encouragement and reinforce exclusive breastfeeding (verbal persuasion). - Period: prenatal. - Frequency: 1 time per two weeks. - Duration: 9 months. | 8 weeks | EBF  BSE^h^ (BSES-SF^i^) |
| Bunik et at[85],  2022, USA | RCT | 469  I_1_:156  I_2_:156  C:157 | - Type：mHealth (app) - The 40 postpartum in-app messages focused on social support strategies, breastfeeding behavioral skills and self-efficacy to address barriers such as latching difficulties, low milk supply and returning to work, with relevant content accessible via app-embedded short videos linked to YouTube. - Period: prenatal. - Frequency: no mention. - Duration:4 months. | 3 months  6 months | EBF  BSE (BSES^j^) |
| Baza et al[114], 2023, USA | RCT | 40  I:20  C:20 | - Type: mHealth (app) - The app provided breastfeeding-related educational content covering its benefits, infant feeding needs timeline, essential maternal supplies, breastfeeding strategies for working mothers, FAQs, and institutional support resources. - Period: prenatal. - Frequency: no mention. - Duration: 6 weeks. | 4-6 weeks | EBF  BSE (BSES-SF) |
| Bilgic et al[84], 2024, Turkey | RCT | 102  I:52  C:50 | - Type: eHealth (telemedicine) - Relies on real-time audio and video transmission technology and online conferencing platforms. Intervention group pregnant women attended two antenatal online sessions on breastfeeding and neonatal care with demonstrations and videos. - Period: prenatal. - Frequency: 1 time per week. - Duration: at least 3 months. | 1 day  1 month  2 months  3 months | EBF |
| Baransel et al[83], 2024, Turkey | RCT | 130  I:65  C:65 | - Type: mHealth (SMS) - Within 24 hours postpartum, participants received breastfeeding and maternal-infant care education, with 6-week mobile messages covering feeding skills, milk management, daily care and emergencies. - Period: postnatal. - Frequency: personalized. - Duration: 6 weeks. | 6 weeks | EBF  BSE (BSES-SF) |
| Candel et al, 2024[117], Spain | RCT | 270  I:136  134 | - Type: mHealth (app) - LactApp provides automated breastfeeding consultation, tracking, expert guidance, reminders and professional lactation support for mothers. - Period: prenatal. - Frequency: no mention. - Duration: 6 months. | 6 months | EBF |
| Çelik et al[87], 2024, Turkey | RCT | 31  I:16  C:15 | - Type: eHealth (telemedicine) - Relies on real-time audio and video transmission, broadband/mobile communication networks, cloud-based data interaction, and instant messaging technologies. Provide remote breastfeeding counseling for adolescents. - Period: postnatal. - Frequency: no mention. - Duration: 8 weeks. | 1 week  4 weeks  8 weeks | EBF  BSE (BSES-SF) |
| Can et al[34], 2025, Turkey | RCT | 90  I:43  C:47 | - Type: eHealth (website) - Based on web server architecture and browser interaction technology. The device-compatible website offers expert-reviewed breastfeeding videos with easy registration, login access and user activity tracking. - Period: prenatal. - Frequency: no mention. - Duration: 8 weeks. | 1 week  8 weeks | EBF  BSE (BSES-SF) |
| Doan et al[89], 2022, Vietnam | RCT | 1266  I:632  C:634 | - Type: mHealth (app) - This app was developed via mixed methods, delivering weekly prenatal and postpartum reminders to promote breastfeeding with offline access. - Period: prenatal. - Frequency: 3 times per week. - Duration: at least 4 months. | 1 month  4 months  6 months | EBF |
| Ertaş et al[49], 2026, Turkey | RCT | 52  I:26  C:26 | - Type: digital device (VR^k^) - Integrating 3D scene modeling, real-time rendering, human posture tracking, and human-computer interaction algorithms, it creates immersive virtual breastfeeding environments. VR devices simulate whole breastfeeding process in hospital and home scenes with immersive interactive operation. - Period: prenatal. - Frequency: no mention. - Duration: 13 weeks. | No mention | BSE (PBSES^l^) |
| Fan et al[90], 2022, China | RCT | 33  I:18  C:15 | - Type: mHealth (app) - Built on a client-server architecture, it relies on community interaction modules. Participants gained online peer support via the app to discuss breastfeeding issues, receiving emotional, informational and appraisal support with professional advice and experience sharing. - Period: postnatal. - Frequency: no mention. - Duration: 6 months. | 1 month  2 months  4 months  6 months | EBF  BSE (BSES-SF) |
| Gilano et al[42], 2025, Ethiopia | RCT | 675  I:337  C:338 | - Type: mHealth (SMS) - The customized Frontline SMS system automatically delivered scheduled Amharic maternal and breastfeeding health messages biweekly for 9 months, with delivery monitoring, automatic retry functions and appointment reminders. - Period: prenatal. - Frequency: 1 time per two weeks. - Duration: 9 months. | 6 months | EBF |
| Henshaw et al[93], 2024, USA | RCT | 116  I:62  C:54 | - Type: mHealth (SMS) - The HHL program provides tablet-based postpartum education for parents, with matched personalized text messages sending targeted reminders and tailored encouragement based on individual coping preferences to both mothers and partners. - Period: postnatal. - Frequency: 1 time per two weeks. - Duration: 9 months. | 6 weeks  6 months | EBF  BSE (BSES-SF) |
| Huang et al[94], 2024, China | RCT | 96  I:48  I:48 | - Type: mHealth (app) - Educational courses focusing on the significance and skills of breastfeeding were provided to participants in the intervention group via a mobile application. - Period: prenatal. - Frequency: no mention. - Duration: 4 months. | 1 month  6 months | EBF  BFK^m^ (BKQ^n^) |
| Harari et al[92], 2018, USA | RCT | 58  I:32  C:26 | - Type: mHealth (SMS) - SMS delivers automated evidence-based breastfeeding education and allows communication with consultants guided by the theory of planned behavior to boost breastfeeding behaviors. - Period: prenatal. - Frequency: 10 times per week. - Duration: 6 months. | 2 weeks | EBF |
| Hmone et al[57], 2023, Myanmar | RCT | 353  I:179  C:174 | - Type: mHealth (SMS) - Send maternal and infant health information to mothers, including breastfeeding-related content. - Period: prenatal. - Frequency: 3 times per week. - Duration: 30-36 weeks. | 1 month  2 months  3 months  4 months  5 months  6 months | EBF |
| KO er al[107], 2025, Kenya | RCT | 412  I:206  C:206 | - Type: mHealth (app) - The app features educational modules, personalized tracking and community support to improve breastfeeding knowledge, attitudes, practices and infant nutrition. - Period: postnatal. - Frequency: 2 times per week. - Duration: 6 months. | 6 months | EBK (BKQ) |
| Koca et al[99], 2026, Turkey | RCT | 64  I:32  C:32 | - Type: eHealth (website) - Built on a client-server architecture, it relies on community interaction modules, instant messaging interfaces, and cloud databases to enable online user discussions, peer experience sharing, and professional advice exchange. All participants' inquiries are answered online by the same professional breastfeeding consultant to ensure care continuity and deliver tailored support as needed. - Period: postnatal. - Frequency: 7 times per week. - Duration: 6 months. | 1 month  2 months  4 months  6 months | EBF |
| Kilic et al[122], 2024, Iran | RCT | 66  I:35  C:31 | - Type: digital device (VR) - VR (Shinecon 3D Virtual Reality) was used for mothers to be able to watch a breastfeeding video after cesarean section delivery. - Period: postnatal. - Frequency: no mention. - Duration: 24 hours. | 4 hours  24 hours | BSE (BSES-SF) |
| Karaahmet et al[97], 2022, Turkey | RCT | 151  I:76  C:75 | - Type: eHealth (telemedicine) - Provide online remote consultation services for cesarean-section mothers to resolve breastfeeding-related problems. - Period: postnatal. - Frequency: no mention. - Duration: 6 months. | 1 month  6 months | EBF  BSE (BSES-SF) |
| Li et al[43], 2024, China | RCT | 290  I:162  C:128 | - Type: mHealth (app) - This function is supported by a cross-platform application framework, video streaming technology, and cloud services. Watch videos via the application covering healthcare, disease prevention and health promotion. - Period: prenatal. - Frequency: no mention. - Duration: 9 months. | 6 months | EBF |
| Moncada et al[104], 2025, Colombia | RCT | 58  I:29  C:29 | - Type: digital device (VR) - These tools included a mixed reality headset, silicone breast model, and infant doll to simulate proper latching and positioning techniques. The intervention also incorporated immersive video content of breastfeeding scenarios, displayed through the VaR VR Video Player application, and a neonatal simulator (infant mannequin) to enhance the realism and interactivity of the training. - Period: prenatal. - Frequency: personalized. - Duration: 1 week. | 1 week | EBF  BSE (BSES-SF) |
| Magalhães et al[100], 2023, USA | RCT | 41  I:19  C:22 | - Type: mHealth (SMS) - This intervention is supported by SMS gateway interfaces and a personalized content delivery engine. Personalized SMS sent by IBCLCs informs mothers of milk sodium levels and offers tailored breastfeeding guidance accordingly. - Period: postnatal. - Frequency: 4 times per week. - Duration: 2 weeks. | 1 month  3 months | EBF |
| Mukunya et al[105], 2025, Uganda | RCT | 1717  I:904  C:813 | - Type: mHealth (SMS) - Automated weekly local-language SMS supplemented peer counseling, covering childbirth preparation, early breastfeeding initiation and postnatal care, to reinforce key health guidance and remind expectant mothers. - Period: prenatal. - Frequency: 1 time per week. - Duration:1 month. | 1 day  1 month | EBF |
| Metin et al[101], 2024, Turkey | RCT | 80  I:40  C:40 | - Type: eHealth (video) - using video shooting and post-production technology, combined with breast and infant model demonstration techniques. Researchers created an educational breastfeeding video with breast and infant models showing initial latching, holding and breast care for pregnant participants. - Period: prenatal. - Frequency: 1 time per week. - Duration: 3 weeks. | 3 weeks | BSE (PBSES) |
| Miremberg et al[102], 2022, Israel | RCT | 197  I：97  C：100 | - Type：mHealth (app) - The application was used mainly for communication. In addition, it included information about the study, team, and information regarding lactation and the possible emotional challenges expected after giving birth. - Period: postnatal. - Frequency: no mention. - Duration: 6 months. | 2 weeks  6 weeks  3 months  6 months | EBF |
| Ni et al[95], 2025, China | RCT | 109  I:55  C:54 | - Type: mHealth (app) - Personalized feedback algorithms. The app monitors breastfeeding duration and frequency via auto or manual recording, providing personalized feeding and nutrient feedback for mothers. - Period: prenatal. - Frequency: personalized. - Duration: 2 months. | 1 month  2 months | EBF |
| Pilus et al[103], 2022, Malaysia | RCT | 172  I:86  C:86 | - Type: mHealth (app) - This intervention leverages instant messaging platform APIs, group interaction technology, and message delivery engines. The WhatsApp group function was used to distribute information, concerns, and issues; clarify any misunderstandings about breastfeeding practice; and provide a reminder to all participants. - Period: postnatal. - Frequency: 1 time per week. - Duration: 7-10 weeks. | 1 month  3 months | EBF  BSE (BSES-SF)  BFK (BKQ) |
| Pooia et al[91], 2026, India | RCT | 128  I:64  C:64 | - Type: eHealth (video) - Conducted an individualized education session through a 2-minute 25-second video in Kannada demonstrating breastfeeding technique. - Period: prenatal. - Frequency: no mention. - Duration: 6 months. | 2 months  4 months  6 months | EBF  BSE (BSES-SF) |
| Raj et al[109], 2025, India | RCT | 120  I:60  C:60 | - Type: mHealth (app) - This intervention delivers breastfeeding education via videos embedded within the application, supported by mobile app client architecture and video streaming playback technology. - Period: postnatal. - Frequency: no mention. - Duration: 1 day. | 2 months  4 months  6 months | EBF  BSE (BSES-SF) |
| Ramadan et al[110], 2025, Saudi Arabia | RCT | 200  I:100  C:100 | - Type：eHealth (telemedicine) - It adopts real-time audio and video transmission, cloud meeting interaction technology, and remote online assessment modules. Participants received structured lactation guidance via Zoom; nurses assessed latching and gave personalized breastfeeding advice remotely. - Period: postnatal. - Frequency: no mention. - Duration: 1 month. | 1 hour  1 week  1 month  3months | EBF  BSE (BSES-SF) |
| Sosanya et al[116], 2025, Nigeria | RCT | 216  I:108  C:108 | - Type: mHealth (app) - The offline Baby Thrive app in English/Hausa teaches breastfeeding and complementary feeding via games, recipes and guidance. - Period: postnatal. - Frequency: no mention. - Duration: 6 months. | 6 months | BFK (BKQ) |
| Seyyedi et al[115], 2021, Iran | RCT | 80  I:40  C:40 | - Type: mHealth (app) - Built on mobile application architecture, cloud content distribution and structured knowledge base technology. The app educates mothers on breastfeeding, complementary feeding, pumping, problem-solving and illness-related lactation queries. - Period: postnatal. - Frequency: no mention. - Duration: 3 months. | 3 months | BSE (BSES)  BFK (KAP^o^) |
| Seddighi et al[113], 2022, Iran | RCT | 187  I:96  C:91 | - Type: mHealth (app) - Adopting mobile application architecture, structured knowledge base. The app offers breastfeeding education covering benefits, techniques, common problems, safe milk handling, and medication safety for mothers. - Period: postnatal. - Frequency: no mention. - Duration: 8 weeks. | 3 months | BSE (BSES-SF) |
| Şimsek et al[35], 2024, Turkey | RCT | 72  I:36  C:36 | - Type: eHealth (telemedicine) - Supported by real-time remote communication technology, cloud-based online interaction modules and standardized curriculum content distribution technology. Intervention group received remote online breastfeeding guidance, emotional support and systematic three-day standardized lactation education. - Period: prenatal. - Frequency: 1 time per 3 days. - Duration: 6 months. | 1 day  1 week  3 months  6 months | BSE (BSES-SF) |
| Wong et al[119], 2025, China | RCT | 146  I:75  C:71 | - Type: eHealth (telemedicine) - Supported by real-time audio and video communication technology, cloud-based online interaction and individualized customized coaching modules, online one-to-one personalized postnatal breastfeeding coaching is provided. - Period: prenatal. - Frequency: personalized. - Duration: 3 months. | 2 months  6 months | EBF  BSE (BSES-SF) |
| Wen et al[118], 2020, Australia | RCT | 1155  I_1_:384  I_2_:385  C:386 | - Type: mHealth (SMS) - Provide education on infant feeding practices to mothers via text messages. - Period: prenatal. - Frequency: 2 times per week. - Duration: 4 weeks. | 6 months  12 months | EBF |
| Wu et al, 2020[56], China | RCT | 319  I:161  C:158 | - Type: mHealth (app) - Supported by instant messaging platform interface, stage-based intelligent content pushing technology, online quiz module, growth chart data management and community forum interaction technology. The module delivers WHO-based breastfeeding education via WeChat with stage-tailored weekly messages, plus quizzes, growth charts and an online forum for mothers. - Period: prenatal. - Frequency: 3 times per week. - Duration: 19 weeks. | 0-1 month  2-3 months  4-5 months | EBF |
| Yikar et al[121], 2026, Turkey | RCT | 80  I:40  C:40 | - Type: mHealth (app) - The app educates mothers on postpartum changes, comfort, breastfeeding self-efficacy, positions, and common lactation issues. - Period: postnatal. - Frequency: no mention. - Duration: 6 weeks. | 1 week  6 weeks | BSE (BSES-SF) |
| Zhu et al[120], 2025, China | RCT | 104  I:55  C:49 | - Type: digital device (VR) - Supported by VR virtual reality technology, 3D scene modeling, labor process simulation engine, human-computer interaction sensing technology and emergency scenario simulation algorithm. VR content includes: (1) familiarization with the delivery room environment; (2) three-dimensional simulation of labor stages, including uterine contractions, cervical dilation and fetal descent; (3) interactive practice of birthing positions and breathing techniques; (4) simulation of potential emergency situations. - Period: prenatal. - Frequency: no mention. - Duration: 5 months. | 42 days | EBF |
| Aram et al[82], 2025, Iran | QES^p^ | 81  I:41  C:40 | - Type: mHealth (app) - The app provides breastfeeding education covering its importance, formula risks, lactation assessment, milk expression, storage and common problem management. - Period: postnatal. - Frequency: at least 1 time per week. - Duration: 8 weeks. | 2 months | EBF |
| Alahmed et al[80], 2024, Saudi Arabia | QES | 290  I:146  C:144 | - Type: eHealth (website) - Supported by web architecture, cloud content publishing and network communication technology. Provide them with basic knowledge about breastfeeding through the website. - Period: prenatal. - Frequency: no mention. - Duration: 1 month. | 1 month | EBF |
| Gürkan et al[86], 2024, Turkey | QES | 73  I:37  C:36 | - Type: eHealth (website) - The website consists of three educational units cover breast milk benefits, effective breastfeeding skills and lactation problems with audios, slides and videos. - Period: postnatal. - Frequency: no mention. - Duration: 15 days. | 15 days | BSE (BSES-SF)  BFK (BBKT^q^) |
| Jiang et al[96], 2015, China | QES | 582  I:281  C:301 | - Type: mHealth (SMS) - Mobile communication networks. Send participants text messages regarding breastfeeding and improving infant feeding practices. - Period: prenatal. - Frequency: 1 time per week. - Duration: 66 weeks. | 4 months  6 months | EBF |
| Karimi et al[88], 2025, Iran | QES | 70  I:35  C:35 | - Type: eHealth (website) - Breastfeeding educational content is uploaded on the website in the form of educational videos and podcasts, and mothers are reminded to view it via the app. - Period: postnatal. - Frequency: 2 times per week. - Duration: 3 weeks. | 3 weeks | BSE (BSES-SF) |
| Nakamura et al[106], 2025, Japan | QES | 23  I:8  C:14 | - Type: eHealth (AI) - Adopts statistical modeling and intelligent learning algorithms. Mothers in the intervention group took close-up nipple photos via an offline smartphone. Deep learning classified nipple conditions into mild, moderate and severe, with targeted breastfeeding feedback provided accordingly. - Period: prenatal. - Frequency: no mention. - Duration: 12 weeks. | 1 month | EBF  BSE (BSES-SF) |
| Ozcoban et al[108], 2025, Turkey | QES | 110  I:55  C:55 | - Type: eHealth (telemedicine) - Relies on real-time audio and video transmission. Provide remote consultation and support for mothers, especially through a hotline for breastfeeding and newborn care. - Period: prenatal. - Frequency: no mention. - Duration: 42 days. | 7-15 days  35-42 days | BSE (BSES-SF) |
| Rostamgohari et al[111], 2025, Iran | QES | 74  I:37  C:37 | - Type: digital device (VR) - Combined with three-dimensional scene modeling, real-time image rendering, human posture tracking and human-computer interaction algorithms, an immersive virtual breastfeeding environment is constructed. - Period: postnatal. - Frequency: no mention. - Duration: 1 week. | 1 week | BSE (BSES-SF) |
| Sarimin et al[112], 2024, Indonesia | QES | 100  I:50  C:50 | - Type mHealth (app) - Based on the client-server model：ASIPku app assists working mothers with exclusive breastfeeding via guidance, pumping reminders, resources and professional online consultation. - Period: prenatal. - Frequency: no mention. - Duration: 6 months. | 6 months | EBF |
| Yildiz et al[98], 2025, Turkey | QES | 60  I:30  C:30 | - Type: eHealth (AI) - React Native-based application integrates ChatGPT API (Natural Language Processing) to provide intelligent responses and personalized guidance to postpartum mothers. - Period: prenatal. - Frequency: 3 times per day. - Duration:10 days. | 4 hours  24 hours  7 days  10 days | BSE (BSES-SF) |

^a^RCT: randomized controlled trial. ^b^I: intervention group. ^c^C: control group. ^d^mHealth: mobile health. ^e^EBF: exclusive breastfeeding. ^f^eHealth: electronic health. ^g^SMS: Short Message Service. ^h^BSE: Breastfeeding Self-Efficacy. ^i^BSES-SF: Breastfeeding Self-Efficacy Scale–Short Form. ^j^BSES: Breastfeeding Self-Efficacy Scale. ^k^VR: Virtual Reality. ^l^PBSES: Prenatal Breastfeeding Self-Efficacy Scale. ^m^BFK: Breastfeeding knowledge. ^n^BKQ: Breastfeeding Knowledge Questionnaire. ^o^KAP: knowledge, attitude, and practice. ^p^QES: Quasi-experimental study. qBBKT: Breastmilk and Breastfeeding Knowledge Test.
