# Supplementary material for: Effects of Digital Health Interventions on Breastfeeding Rates, Self-Efficacy, and Knowledge: Systematic Review and Meta-Analysis
Source: J Med Internet Res. 2026 Aug 12;28:e92184. doi: 10.2196/92184 (PMC13519512; doi:10.2196/92184)
Supplement: Supplementary file 5 [file jmir_v28i1e92184_app5.docx]

Table 2.

Summary of Evidence Quality Ratings for the Effects of Digital Health Interventions on Breastfeeding Outcomes (Exclusive Breastfeeding Rate, Breastfeeding Self-Efficacy, and Breastfeeding Knowledge) based on the GRADEᵃ Framework.

| Outcome | Studies (n, k) | Sample size (N) | | Effect size (95% CI) | Risk of bias | Inconsistency | Indirectness | Imprecision | Other considerations | Certainty |
| --- | --- | --- | --- | --- | --- | --- | --- | --- | --- | --- |
|  |  | Intervention | Control |  |  |  |  |  |  |  |
| Exclusive breastfeeding rate at <3months | 30 | 2026/2794 (72.5%) | 1503/2664 (56.4%) | RR^b^ 1.21 (1.13 to 1.29) | not serious | serious^c^ | not serious | not serious | none | ⨁⨁⨁◯ Moderate |
| Exclusive breastfeeding rate at 3-6months | 15 | 739/1473 (50.2%) | 493/1369 (36.0%) | RR 1.54 (1.29 to 1.85) | not serious | serious^d^ | not serious | not serious | none | ⨁⨁⨁◯ Moderate |
| Exclusive breastfeeding rate at ≥6months | 16 | 1115/2451 (45.5%) | 719/2308 (31.2%) | RR 1.34 (1.25 to 1.43) | not serious | serious^e^ | not serious | not serious | potential small-study effects^f^ | ⨁⨁◯◯ Low |
| Breastfeeding self-efficacy | 18 | 1473 | 1354 | SMD 0.41 (0.04 to 0.78) | not serious | serious^g^ | not serious | not serious | none | ⨁⨁⨁◯ Moderate |
| Breastfeeding knowledge | 5 | 515 | 513 | SMD 1.69 (0.54 to 2.84) | not serious | serious^h^ | not serious | serious^i^ | none | ⨁⨁◯◯ Low |

ᵃGRADE:Grading of Recommendations, Assessment, Development, and Evaluation.

^b^RR:relative risk

^c^Inconsistency:significant heterogeneity existed among studies (*I*^2^=84%), indicating substantial variation in intervention effects across studies, thus the quality was downgraded by one level.

^d^Inconsistency:the original pooled results showed high heterogeneity (I^2^=75%) , thus the quality was downgraded by one level.

^e^Inconsistency:the original pooled results showed high heterogeneity (I^2^=86%) , and 95% PI spans a wide range, thus the quality was downgraded by one level.

^f^Other considerations:Egger’s test indicated potential small-study effects (*P* = .006). Although the results remained robust following trim-and-fill adjustment, substantial heterogeneity persisted, leading to a one-level downgrade in evidence quality per standard GRADE procedures.

^g^Inconsistency:significant heterogeneity existed among studies (*I*^2^=95%), and the prediction interval (95% PI –1.17 to 2.51) crossed the null line, indicating substantial variation in intervention effects across studies, thus the quality was downgraded by one level.

^h^Inconsistency:the original pooled results showed high heterogeneity (*I*^2^=98%) and the prediction interval crossed the zero line, thus the quality was downgraded by one level.

^i^Imprecision:the number of included studies was small (k=6), the sample size was insufficient, and the 95% CI crossed the null line, indicating a risk of imprecision, therefore the quality was downgraded by one level.
