# Supplementary material for: Effects of Digital Health Interventions on Breastfeeding Rates, Self-Efficacy, and Knowledge: Systematic Review and Meta-Analysis
Source: J Med Internet Res. 2026 Aug 12;28:e92184. doi: 10.2196/92184 (PMC13519512; doi:10.2196/92184)
Supplement: Supplementary file 6 [file jmir_v28i1e92184_app6.docx]

**Multimedia Appendix 6**

Figure S1. (A) Forest plot of subgroup analysis of intervention type for exclusive breastfeeding rates at <3 months. (B) Forest plot of subgroup analysis of intervention type for exclusive breastfeeding rates at 3-6 months. (C) Forest plot of subgroup analysis of intervention type for exclusive breastfeeding rates at ≥6 months.

**(A)**

**(B)**

**(c)**

Figure S2. (D) Forest plot of subgroup analysis of intervention period for exclusive breastfeeding rates at <3 months. (E) Forest plot of subgroup analysis of intervention period for exclusive breastfeeding rates at 3-6 months. (F) Forest plot of subgroup analysis of intervention period for exclusive breastfeeding rates at ≥6 months.

**(D)**

**(E)**

**(F)**

Figure S3. (G) Forest plot of subgroup analysis of intervention duration for exclusive breastfeeding rates at <3 months. (H) Forest plot of subgroup analysis of intervention duration for exclusive breastfeeding rates at 3-6 months. (I) Forest plot of subgroup analysis of intervention duration for exclusive breastfeeding rates at ≥6 months.

**(G)**

**(H)**

**(I)**

Figure S4. (J) Forest plot of subgroup analysis of intervention frequency for exclusive breastfeeding rates at <3 months. (K) Forest plot of subgroup analysis of intervention frequency for exclusive breastfeeding rates at 3-6 months. (L) Forest plot of subgroup analysis of intervention frequency for exclusive breastfeeding rates at ≥6 months.

**(J)**

**(K)**

**(L)**

Figure S5. (M) Forest plot of subgroup analysis of national income level for exclusive breastfeeding rates at <3 months. (N) Forest plot of subgroup analysis of national income level for exclusive breastfeeding rates at 3-6 months. (O) Forest plot of subgroup analysis of national income level for exclusive breastfeeding rates at ≥6 months.

**(M)**

**(N)**

**(O)**

Figure S6. (P) Forest plot of subgroup analysis of gestational age for exclusive breastfeeding rates at <3 months.

**(P)**

Figure S7. (Q) Forest plot of subgroup analysis of maternal age for exclusive breastfeeding rates at <3 months.

**(Q)**

Figure S8. (R) Forest plot of subgroup analysis of mode of delivery for exclusive breastfeeding rates at <3 months. (S) Forest plot of subgroup analysis of mode of delivery for exclusive breastfeeding rates at 3-6 months. (T) Forest plot of subgroup analysis of mode of delivery for exclusive breastfeeding rates at ≥6 months.

**(R)**

**(S)**

**(T)**

Figure S9. Forest plot of subgroup analysis of intervention type for breastfeeding self-efficacy.

Figure S10. Forest plot of subgroup analysis of intervention period for breastfeeding self-efficacy.

Figure S11. Forest plot of subgroup analysis of intervention duration for breastfeeding self-efficacy.

Figure S12. Forest plot of subgroup analysis of intervention frequency for breastfeeding self-efficacy.

Figure S13. Forest plot of subgroup analysis of national income level for breastfeeding self-efficacy.

Figure S14. Forest plot of subgroup analysis of gestational age level for breastfeeding self-efficacy.

Figure S15. Forest plot of subgroup analysis of maternal age level for breastfeeding self-efficacy.

Figure S16. Forest plot of subgroup analysis of mode of delivery level for breastfeeding self-efficacy.

Figure S17. Forest plot of subgroup analysis of fetal number level for breastfeeding self-efficacy.

Figure S18. Forest plot of subgroup analysis of intervention period for breastfeeding knowledge.

Figure S18. Forest plot of subgroup analysis of intervention duration for breastfeeding knowledge.
