# Supplementary material for: Effects of Digital Health Interventions on Breastfeeding Rates, Self-Efficacy, and Knowledge: Systematic Review and Meta-Analysis
Source: J Med Internet Res. 2026 Aug 12;28:e92184. doi: 10.2196/92184 (PMC13519512; doi:10.2196/92184)
Supplement: Supplementary file 7 [file jmir_v28i1e92184_app7.docx]

**Multimedia Appendix 5**

Figure S1. Funnel plots of exclusive breastfeeding rates at < 3 months.

Test result: t = 0.7503, df = 28, p = 0.4593

Figure S2. Funnel plots of exclusive breastfeeding rates at 3-6 months.

Test result: t = 1.0311, df = 11, p = 0.3246

Figure S3. Funnel plots of exclusive breastfeeding rates at ≥6 months.

Test result: t = 3.1716, df = 15, p = 0.0063

Figure S4. Funnel plots of breastfeeding self-efficacy.

Test result: t = 1.2507, df = 26, p = 0.2222

Figure S5. Funnel plots of breastfeeding knowledge.

Test result: t = 1.0147, df = 4, p = 0.3676
