# Supplementary material for: Effects of Digital Health Interventions on Breastfeeding Rates, Self-Efficacy, and Knowledge: Systematic Review and Meta-Analysis
Source: J Med Internet Res. 2026 Aug 12;28:e92184. doi: 10.2196/92184 (PMC13519512; doi:10.2196/92184)
Supplement: Supplementary file 8 [file jmir_v28i1e92184_app8.docx]

**Multimedia Appendix 4**

Figure S1. Sensitivity analysis of exclusive breastfeeding rates at < 3 months.

Figure S2. Sensitivity analysis of exclusive breastfeeding rates at 3-6 months.

Figure S3. Sensitivity analysis of exclusive breastfeeding rates at ≥6 months.

Figure S4. Sensitivity analysis of breastfeeding self-efficacy.

Figure S5. Sensitivity analysis of breastfeeding knowledge.
